# Immunodeficiency-Related Vaccine-Derived Poliovirus (iVDPV) Infections: A Review of Epidemiology and Progress in Detection and Management

**DOI:** 10.3390/pathogens13121128

**Published:** 2024-12-20

**Authors:** Concepcion F. Estivariz, Elisabeth R. Krow-Lucal, Ondrej Mach

**Affiliations:** 1 U.S. Centers for Disease Control and Prevention, 1600 Clifton Road, Atlanta, GA 30033, USA; liz.krowlucal@gatesfoundation.org; 2 World Health Organization Headquarters, Av Appia 10, 1211 Geneva, Switzerland; macho@who.int

**Keywords:** immunodeficiency disorders, poliovirus, vaccine-derived poliovirus, vaccine-associated paralytic poliomyelitis

## Abstract

Individuals with certain primary immunodeficiency disorders (PID) may be unable to clear poliovirus infection after exposure to oral poliovirus vaccine (OPV). Over time, vaccine-related strains can revert to immunodeficiency-associated vaccine-derived poliovirus (iVDPVs) that can cause paralysis in the patient and potentially spread in communities with low immunity. We reviewed the efforts for detection and management of PID patients with iVDPV infections and the epidemiology through an analysis of 184 cases reported to the World Health Organization (WHO) during 1962–2024 and a review of polio program and literature reports. Most iVDPV patients (79%) reported in the WHO Registry were residents in middle-income countries and almost half (48%) in the Eastern Mediterranean Region. Type 2 iVDPV was most frequently isolated (53%), but a sharp decline was observed after the switch to bivalent OPV in 2016, with only six cases reported during 2017–2024 compared to 63 during 2009–2016. Patients with common variable immunodeficiency have longer excretion of iVDPV than with other PID types. Implementation of sensitive sentinel surveillance to detect cases of iVDPV infection in high-risk countries and offer antiviral treatment to patients is challenged by competition with other health priorities and regulatory hurdles to the compassionate use of investigational antiviral drugs.

## 1. Introduction

The use of oral poliovirus vaccine (OPV) worldwide in childhood immunization and vaccination campaigns has been the main strategy of the Global Polio Eradication Initiative (GPEI). This approach has reduced wild poliovirus (WPV) circulation by 99.9%, from >350,000 estimated cases in 125 countries in 1988 to 12 cases in two countries in 2023 [1]. The live, attenuated Sabin strains contained in OPV multiply in oropharyngeal and gastrointestinal mucosae, mimicking natural infection, and induce humoral and mucosal immunity. Upon new exposure to wild or vaccine poliovirus, humoral immunity protects against paralysis whereas mucosal immunity reduces viral replication and excretion through stools or oropharyngeal secretions, thus interrupting person-to-person transmission [2,3]. Additionally, OPV is easy to administer in mass campaigns and vaccine virus can indirectly vaccinate close contacts, which enhances field effectiveness.

However, the genetic instability of Sabin strains leads them to mutate and recombine with other enteroviruses, whereby they may re-acquire the fitness of wild strains [4,5,6]. Reversion of key mutations associated with neurovirulence can occur shortly after receiving OPV and on rare occasions may result in vaccine-associated poliomyelitis (VAPP) in recipients or close contacts [7,8]. Prolonged replication within one individual or sustained person-to-person transmission within a community with low immunity allows Sabin-related strains to evolve to vaccine-derived poliovirus (VDPVs) which have re-acquired the neurovirulence and transmissibility of wild poliovirus strains [5,6,9,10]. In areas with low population immunity, outbreaks of infections due to circulating VDPVs (cVDPVs) require several immunization campaigns to stop transmission and prevent the establishment of endemic poliovirus transmission [11]. Since 2017, the reported numbers of paralytic polio cases caused by cVDPV have exceeded cases caused by WPV, with a total of 419 WPV cases and 3794 cVDPV cases reported during 2017–2023 (data as of 30 September 2024) [1]. The risks of VAPP and cVDPV outbreaks associated with OPV led the GPEI to conclude that cessation of OPV use is essential to maintain a polio-free world after the eradication of WPV [12].

Individuals with certain primary immunodeficiencies (PID) that severely affect B-cell function may be unable to clear vaccine poliovirus after receiving OPV or having been in contact with a vaccinee, therefore becoming asymptomatic carriers for months or years. With prolonged replication in these individuals, Sabin-related strains evolve to immunodeficiency-associated VDPVs (iVDPVs). Sabin strains or iVDPVs may cause paralytic polio at any time in the immunodeficient individual, resulting in an estimated risk of paralytic polio associated with OPV about 3000-fold higher than that observed for immunocompetent individuals [7,13]. PID patients shedding iVDPVs may also theoretically spread poliovirus in communities with low immunity, posing a potential threat for the re-introduction of poliovirus and outbreaks after the eradication of WPV and cessation of OPV use [14].

To better understand and mitigate the risks posed by iVDPV excretors to global polio eradication, the GPEI has maintained a registry of immunodeficient cases with prolonged shedding of poliovirus or iVDPV since 1961. During the early 2000s, GPEI supported studies to assess the prevalence of prolonged excretion of poliovirus among patients with different types of immunodeficiency disorders in a variety of countries. In the last decade, GPEI has pursued the introduction of sentinel surveillance for the detection of poliovirus among patients with certain PIDs in several higher-middle and lower-middle income countries considered at higher risk for iVDPV emergence. In addition, since the early 2000s, GPEI partners have supported the development of antivirals and monoclonal antibodies that can be used to treat chronic poliovirus infections.

This report summarizes current knowledge on the clinical and epidemiological characteristics of poliovirus infection in immunodeficient individuals. We reviewed the biomedical literature on prevalence studies and case reports published to date and performed an updated epidemiological analysis of patients with iVDPV infection reported to the WHO Registry during 1962–2024. In addition, we describe current GPEI efforts to detect and treat PID patients with iVDPV infection to interrupt shedding and prevent potential community transmission.

## 2. Materials and Methods

### 2.1. Study Population

This study analyzes cases reported to the iVDPV Data Registry maintained by the World Health Organization (WHO) from 1962 to June 2024 and cases identified through a literature search. The main sources of cases reported in the WHO iVDPV Registry were focal points for polio surveillance or representatives of the Global Poliovirus Laboratory Network (GPLN) at country and regional levels. Initially, most of the cases were identified through surveillance for Acute Flaccid Paralysis (AFP). Since 2010, a high number of cases with or without paralysis were identified during cross-sectional studies or pilot surveillance projects that screened patients with suspected or diagnosed PID for poliovirus infection.

To identify possible cases missing in the WHO database, we conducted a search of articles published in peer-reviewed journals. We used previous systematic reviews for reference mining on patients with iVDPV infection up to 2016 [5,15,16]. For the years 2014–2024, we conducted a literature search in PubMed using key search terms: VDPV, iVDPV, immunodeficiency, immunocompromised, polio, and “vaccine-associated paralytic poliomyelitis”.

Our definition for an iVDPV case for analysis was a laboratory-confirmed VDPV infection in a person of any age with a primary humoral or combined humoral and cellular immunodeficiency disorder. Six cases in the WHO iVDPV Data Registry as of 24 July 2024 were excluded after confirmation of duplicate entry, and two cases were excluded because there was insufficient information to confirm iVDPV (i.e., no genetic sequencing performed).

Testing for poliovirus in stool, oropharyngeal swabs, or other specimens from iVDPV cases was mostly performed in laboratories that are members of the WHO Global Polio Laboratory Network (GPLN), using standardized protocols [17]. Testing usually involves the isolation of poliovirus through growth in cultured cells, followed by an enzyme-linked immunosorbent assay or a reverse transcriptase polymerase chain reaction to identify the serotype and differentiate between wild and vaccine strains. Lastly, sequencing of the genome sequence encoding the major capsid protein, viral protein 1 (VP1), is conducted to distinguish VDPV from Sabin-related strains. Based upon the nucleotide substitution rate of ~1.1% per year in the VP1-coding region, VDPVs are defined as having a VP1 nucleotide divergence from the Sabin strain >1% for types 1 and 3, and >0.6% for type 2. Genetic sequencing of the VP1 region, and in some cases, full poliovirus genome sequencing, allows the assessment of nucleotide divergence between strains and the tracking of pathways of transmission of WPV and VDPVs [5,18].

### 2.2. Statistical Analysis

Epidemiological data available in the database included the patient’s country of residence, patient’s date of paralysis onset, date of first positive iVDPV isolation (and additional samples), patient gender, age at paralysis onset or iVDPV detection, polio serotype, and percentage VP1 divergence from the parenteral Sabin strain. If missing, information on type of PID, presence or absence of paralysis, clinical outcome (dead, alive and stopped excreting, alive and continued excreting at the time of reporting), and date of latest positive iVDPV isolate was supplemented with information from case reports in scientific journals and consultation with surveillance focal points, when possible.

Descriptive analysis on demographic and clinical characteristics of iVDPV cases was conducted using R statistical software (v3.4; R Core Team 2018). The duration of excretion under observation was calculated assuming a nucleotide substitution rate of ~1.1% VP1 divergence from Sabin strain per year [18]. Prolonged infection is defined as poliovirus excretion lasting > 6 months and chronic infection as poliovirus excretion > 5 years. Countries are classified by World Bank income level criteria for designation as low, lower-middle, upper-middle, and high income.

## 3. Results

### 3.1. Immune Response to OPV and Emergence of VDPVs

#### 3.1.1. Immune Response in Immunocompetent Individuals

Serum neutralizing antibodies are the major determinants of protection against poliomyelitis, although cellular immunity also plays a role [19,20]. Immunity for each of the three poliovirus types is independent although there is some cross-reactivity, especially in early immune responses.

Following natural infection or OPV administration, polioviruses multiply locally the in tonsils, pharyngeal and lower gastrointestinal mucosae, and in gut-associated lymphoid tissue, and then spread to systemic lymphoid tissues and other sites for secondary multiplication, causing transient viremia. Therefore, poliovirus may be detected in oropharyngeal secretions for 1 to 2 weeks and in stools for about 4–8 weeks [21,22,23]. In immunocompetent individuals, viral replication induces gut-associated CD4+ T helper cells and locally produced cytokines, which drive the development of B-cells that produce poliovirus-neutralizing secretory immunoglobulin (Ig) A. Secretory IgA is transported across the intestinal epithelium into the gut lumen, and its appearance 1–3 weeks after OPV administration is associated with a progressive reduction in viral shedding [24,25]. Cytotoxic CD4 and CD8 T-cell responses also appear to be involved in the clearance of virus-infected cells [20].

Serum IgM and IgG are detected approximately 7–10 days after vaccination. IgM disappears within 2 months whereas IgG persists for years, with titers boosted upon new exposure to live or inactivated poliovirus [26,27]. Even when serum-neutralizing antibody titers go below the limits of detection, immunological memory in B-cells appears to provide life-long protection against paralysis [28]. Mucosal intestinal immunity, provided mostly by secretory IgA, prevents viral replication upon a new infection or vaccine dose, as shown by challenge studies [21,23,29]. However, mucosal immunity is only partial in that subsequent vaccine doses has resulted in the shedding of vaccine poliovirus strains in a portion of immune individuals, although for shorter time and in lower amount than in susceptible individuals. Mucosal immunity also wanes within a few years in the absence of new exposure to poliovirus [3,21,23,29].

#### 3.1.2. Vaccine-Associated Paralysis (VAPP) and Vaccine-Derived Poliovirus (VDPV)

Paralytic poliomyelitis results when poliovirus reaches and destroys motor-neurons in the anterior horn of the spinal cord or in the brainstem, following viremia or through retrograde axonal transport, before neutralizing antibodies have reached protective levels. With WPV, the ratio of paralysis to infection among unvaccinated individuals ranges from 1:200 to 1:2000, with the highest neurovirulence for type 1 and the lowest for type 2 [2,30].

Replication of vaccine-related poliovirus strains in the gastrointestinal tract for several weeks allows genomic changes that modify the Sabin-related strains’ genome at a rate of ~1.1% nucleotide substitution per year in the region coding (VP1) [5,18,22]. Those genomic changes often include the reversion of the key mutations attenuating neurovirulence and virus fitness to replicate in infected hosts. There is also intertypic recombination among Sabin-related strains, which may eliminate fitness-decreasing mutations in the parental strain [4,31]. Reversion of neurovirulence sites likely occurs faster for types 2 and 3 than type 1 because they have fewer attenuating substitutions, with post-vaccination shedding of neurovirulent strains observed in up to 75% of infants vaccinated with Sabin type 2 [5,18,32].

Mutations in key attenuated sites in Sabin-related strains are associated with the development of vaccine-associated paralytic poliomyelitis (VAPP) following vaccination. The clinical presentation of VAPP is equivalent to poliomyelitis caused by WPV. Isolation of a vaccine-like virus and/or a temporal link with OPV exposure is usually required for categorization [7,8]. Among immunocompetent patients, recipient VAPP case patients’ symptoms usually start 4 to 30–40 days after receiving OPV and 5 to 75 days after exposure to a vaccinee in a contact VAPP case. In the United States, VAPP risk was estimated at about one case per 900,000 for the first OPV doses distributed and declined sharply with subsequent doses in the series [7,8].

Prolonged person-to person transmission of Sabin-related strains in a community or prolonged replication of Sabin strains within a single individual with an immunodeficiency disorder allows the accumulation of reverting mutations and recombination with other enterovirus that increase their fitness to multiply and spread. These vaccine-derived polioviruses (VDPVs) have, by definition, a divergence from the parental Sabin strain in the VP1-coding region of the genome of >1% for types 1 and 3 or >0.6% for type 2 [33] and are assumed to have the transmissibility and neurovirulence of WPV of the same serotype [5,6,9,10]. VDPVs have been classified into three categories based upon their origin status and public health importance. Circulating (cVDPVs), for which community circulation has been demonstrated, emerge in communities with low mucosal immunity to poliovirus because of low vaccination coverage and an absence of WPV circulation. Immunodeficiency-associated poliovirus (iVDPV) is isolated from individuals with PIDs following prolonged replication of Sabin-related strains within the individual. Lastly, ambiguous VDPV (aVDPV) includes isolated detections in samples from a child with AFP or in an environmental sample without demonstration of community circulation [33].

Detection of cVDPVs in a previously polio-free area requires a public health response similar to that used to control WPV outbreaks of the same type, whereas detection of a patient with iVDPV in the absence of community circulation requires case management and follow up [1].

#### 3.1.3. Primary Immunodeficiencies

PIDs result from more than 400 genetic errors regarding functions intrinsic to the cells and tissues of the immune system [34]. Patients with PID have increased susceptibility to infections; recurrent pyogenic and viral infections typically occur with defects of humoral (B-cell) immunity and opportunistic infections with defects of cell-mediated immunity. Patients with PID also have a high risk of autoimmune diseases, allergies, or malignancy. Because the generation of poliovirus-specific neutralizing antibodies plays a major role in clearing enterovirus infections, individuals with pure B-cell deficiencies and combined B- and T-cell deficiencies have a higher risk for prolonged infection by enteroviruses than PIDs affecting other components of the immune system.

B-cell deficiencies are characterized by very low levels of immunoglobulins and B-cells in blood but normal numbers of lymphoid and circulating T-cells and appropriate response to mitogens. B-cell deficiencies are the most common PIDs, with X-linked agammaglobulinemia (AGG) prevalence estimated at 1 per 700,000 individuals and common variable immunodeficiency (CVID) at around 1 per 50,000 individuals in high-income countries [14,35]. X-linked AGG usually presents at 9–12 months of age with recurrent pyogenic infections such as otitis media, sinusitis or pneumonia, unusual and potentially fatal forms of enterovirus infections, or with chronic diarrhea by parasites such as Giardia lamblia. CVID is a heterogeneous collection of conditions with a primary antibody deficiency of at least two immunoglobulin isotypes, caused by different genetic factors. CVID often presents during the second or third decade of life as recurrent sinopulmonary infections, although some patients have infections by unusual pathogens such as *Pneumocystis carini* or mycobacteriae, or chronic diarrheal disease from enterovirus infections or parasitic infestations. CVID also manifests non-infectious complications including autoimmune diseases, non-infectious gastrointestinal disease, granulomatous inflammation, and increased risk of malignancy. Patients with B-cell deficiencies require lifelong replacement therapy with immunoglobulin, antibiotics for treatment and prevention of infections, and appropriate therapy for noninfectious complications [36,37].

Severe combined immunodeficiency (SCID) has multiple genetic causes but presents with a quite uniform phenotype [34]. By three months of age, infants present with a persistent rash, intractable diarrhea, failure to thrive, and, frequently, pneumonia, with profound lymphopenia and absent thymus. Death may occur very quickly from viral infections or adverse outcomes from live vaccines. Bone marrow transplantation as quickly as possible is the only treatment for severe cases; for less severe forms of combined defects, immunoglobulin replacement therapy and management of infections may be successful [37]. Major histocompatibility complex (MHC) class II molecules expressed on antigen-presenting cells and activated T-cells are important for both cell-mediated and humoral immunity. Genetic defects encoding these sites have been found at higher frequency in North African countries [35]. Children present with severe, protracted diarrhea, pneumonia and severe upper respiratory infections, and failure to thrive. Although less severe than SCID, this PID is fatal by the first or second decade of life, but bone marrow transplantation has resulted in long-term survival [37].

#### 3.1.4. Immune Response to Poliovirus in Immunodeficient Individuals

Early studies in the 1960s showed that only a few individuals with immunodeficiencies had a prolonged poliovirus infection following OPV administration, without evident higher risk of poliomyelitis. Among 30 hypogammaglobulinemic patients who received monovalent OPVs sequentially in 1961, only two individuals presented with prolonged infections: a 3-year-old child who shed type 1 virus after monovalent OPV type 1 administration for about 3.5 years, and a 20-year-old woman who shed type 3 virus for about 23 months. The child’s shedding was terminated by a Shigella infection whereas the adult’s shedding stopped spontaneously [38,39].

As OPV became more widely used, it was confirmed that a small proportion of immunodeficient individuals could have asymptomatic poliovirus infection for months or years following receipt of OPV or exposure to contact vaccinees. Prolonged infection was defined as poliovirus excretion lasting > 6 months and chronic infection as poliovirus excretion > 5 years [2].

With prolonged infection of vaccine poliovirus in immunodeficient individuals, Sabin-related strains evolve to iVDPVs that can be isolated in stools and, occasionally, in oropharyngeal secretions [40]. The iVDPVs are defined by the extent of VP1 nucleotide sequence divergence from their corresponding OPV strain serotype in a similar way as cVDPVs. There are molecular differences between iVDPVs and cVDPVs attributed to the different selective pressures exerted on the virus during prolonged infection within a single individual instead of through multiple individuals [5,6]. Key distinguishing features of iVDPVs from cVDPVs include the presence of multiple lineages within one individual, as polioviruses colonize different areas of the gut that evolve independently, the presence of non-recombinant or vaccine–vaccine recombinant genomes instead of recombinants with other species C enteroviruses, and the presence of more extensive antigen variability than typically observed with cVDPVs of the same age (more non-synonymous nucleotide substitutions vs. synonymous substitutions that code for the same amino acid) [6,41,42,43]. Despite these differences, iVDPVs are similarly assumed to have lost the attenuated phenotypes for virulence and transmissibility as cVDPVs.

Fortunately, only a very small number of individuals with certain immunodeficiencies are at risk of prolonged infection with Sabin-related strains or with further evolved iVDPVs. Although patients with isolated IgA deficiency may eliminate polioviruses less efficiently than normal individuals [44], a multicentric study in the early 2000s confirmed the very low risk of prolonged poliovirus excretion and of paralytic polio by vaccine-related strains among individuals with mild IgG or IgA deficiencies [45]. Several studies have failed to find prolonged excretors of poliovirus in children or adults with T-cell immunodeficiencies secondary to HIV infection [46,47,48,49]. Lastly, the risk also appears to be very small among patients with secondary immunodeficiencies related to cancer or its treatment [46].

Cross-sectional studies conducted in 19 countries during 2008–2021 found only 59 out of 1717 (3%) individuals with B-cell PIDs excreting poliovirus at the time of the study (Table 1). The prevalence of poliovirus shedding reported in these studies ranged from 0–25%, with the highest prevalence observed in a study conducted in Tunisia shortly after an immunization campaign. Only 15 (1%) of the PID patients identified in these studies were shedding iVDPV [45,50,51,52,53,54,55,56,57,58].

Follow-up of immunodeficient patients with iVDPV infection identified through surveillance for acute flaccid paralysis or cross-sectional studies has provided more information about the PIDs at the highest risk for prolonged poliovirus infections. Among the 137 immunodeficient patients with iVDPV infection in the WHO Registry for whom the type of PID was known, 45 patients (33%) had SCID, 23 (17%) had agammaglobulinemia, 22 (16%) had CVID, and 19 (14%) had MHC class II deficiency (Table 2).

A number of immunodeficient individuals die shortly after detection of vaccine-like virus or iVDPV infection from complications related to their PID or to poliomyelitis, whereas other patients clear the poliovirus infection after several months or years, resulting in a very small number with chronic infection (>5 years). A study in the US following 37 immunodeficient individuals who developed paralytic polio during 1975–1997 found that 19% were shedding the virus at 6 months, 14% at 1 year, 5% at 4 years, and 0% by 10 years [61]. Among the 98 iVDPV-infected patients in the WHO Registry with follow-up available, the median duration of iVDPV excretion following detection was 3.7 months (ranging <1 month–26 years) and only 10 cases with excretion above 5 years have been reported between 1962 and 2024 (Supplementary Table S1).

Patients with SCID and CVID appear to have the highest difficulty in clearing iVDPV infection, which suggests a significant role for cell-mediated immunity in mounting an adequate immune response to enterovirus infections [16]. However, as opposed to SCID patients, who often die young from infectious complications or stop excreting after bone marrow transplant [16,36], CVID patients are the group with the highest risk of prolonged asymptomatic infection. The median duration of infection between detection and death or clearing among 22 CVID patients with steady follow-up was 0.63 years (range 0.04–26.42), compared to 0.2 years among patients with SCID, or 0.3 years among patients with agammaglobulinemia (Table 3). Of the 10 patients with identified chronic poliovirus excretion, 8 of them had CVID [39,55,62,63,64,65,66,67,68] (Supplementary Table S1).

In several cases, iVDPV infection was resolved after severe diarrheal disease [39,64,69]. But, other than re-acquisition of immunocompetence with bone marrow transplant [70,71] or treatment with antivirals [66], some cases stop iVDPV excretion after months or years without clear reasons. Unfortunately, we only have anecdotal information on patients’ outcomes following treatment from some of the published case reports because this information is not collected in the WHO Registry.

### 3.2. Epidemiological and Clinical Presentation of Poliovirus Infection in Immunodeficient Patients

#### 3.2.1. Demographic Characteristics of iVDPV Cases Reported to the WHO Registry

**Figure 1:**
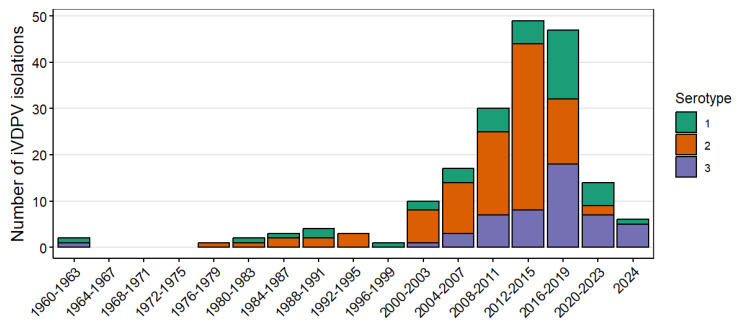
Number of immunodeficiency-associated vaccine-derived poliovirus (iVDPV) isolations reported during 1962–2024 by year of detection and serotype of first positive specimen. The graphic includes 189 isolates in stool specimens from 184 patients with type 1 (n = 42), type 2 (n = 97), and type 3 (n = 50). Five patients with co-infection are counted twice: three patients co-infected with types 1 and 2, one patient with types 2 and 3, and one patient with types 1 and 3.

As of July 2024, the WHO Registry included 184 cases of iVDPV infection detected during 1962–2024. The predominant type of poliovirus in all cases was type 2, detected in 97 (53%) cases, followed by types 3 and 1 detected in 50 (27%) and 42 (23%) of cases, respectively (Table 2). Figure 1 shows the number of iVDPV cases and serotype reported over time since 1962. The number of iVDPV type 2 isolations decreased after the 2016 global switch from trivalent OPV (tOPV) to bivalent OPV (bOPV), from 36 isolations reported during 2012–2015, to 14 during 2016–2019, and 2 during 2020–2023. Two cases, both detected in 2022 in Egypt, have been linked to novel OPV type 2 (nOPV2, genetically more stable in replication than Sabin-strain OPV type 2), despite the distribution of >900 million doses in outbreak response campaigns during 2021–2024 [72]. Isolation of iVDPV types 1 and 3 among reported cases increased during 2016–2019, with 33 isolations reported, compared to 12 and 13 detected in the 2008–2011 and 2012–2015 periods, respectively; the number for both declined during 2020–2023.

**Figure 2:**
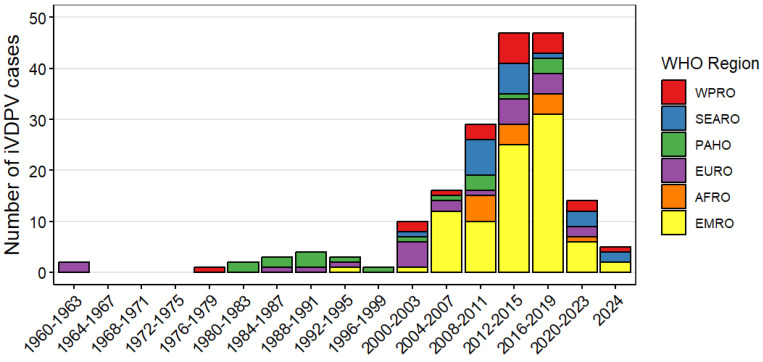
Number of immunodeficiency-associated vaccine-derived poliovirus (iVDPV) cases reported during 1962–2024 by WHO region (N = 184). The x-axis is split in four-year intervals with the total number of cases reported in that year shown as the bar. AFRO—African Region; EMRO—Eastern Mediterranean Region; EURO—European Region; PAHO—Pan-American Health Organization, Region of the Americas; SEARO—South-East Asian Region; WPRO—Western Pacific Region.

**Figure 3:**
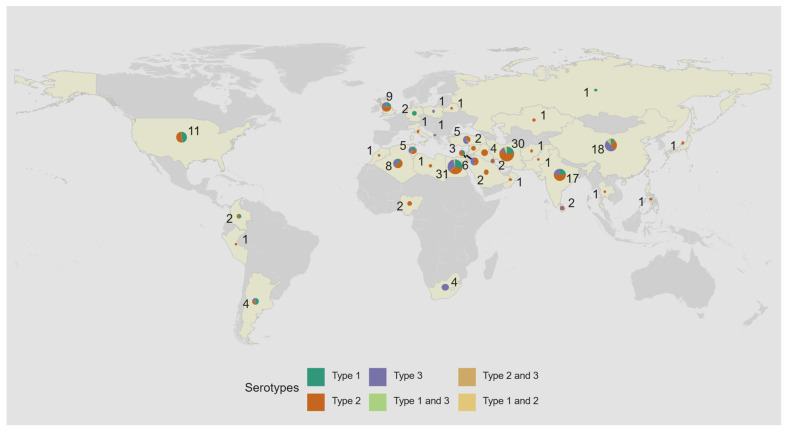
Geographic location of 184 immunodeficiency-associated vaccine-derived poliovirus (iVDPV) cases reported during 1962–2024 by country and serotype. The total number of iVDPV cases by serotype reported over the time period are represented by pie charts proportional to the number of isolations. Five patients had co-infection. Countries that have reported at least one iVDPV case from 1962 to 2024 to date are highlighted in yellow.

Since 2008, there has been a shift in reported iVDPV cases from high income countries in the European and the Americas WHO regions to middle income countries in the Eastern Mediterranean Region (Figure 2 and Table 2). As of July 2024, most of the iVDPV cases reported to the WHO Registry (88, 48%) resided in the Eastern Mediterranean WHO Region. Overall, 77 (42%) cases resided in upper-middle income, 68 (37%) in lower-middle income, 38 (21%) in high income countries, and only 1 (1%) in a low-income country (Table 2). Egypt and Iran, with 31 and 30 cases, respectively, are the countries with the highest number of reported cases, followed by China (22) and India (17), as shown on Figure 3.

Among patients with reported details, males accounted for 103/170 (61%) of iVDPV cases; 83/169 (49%) cases were detected before one year of age (Table 2). At the time of writing this report, 74/184 (40%) patients in the registry had died, 62 (34%) had stopped excreting, and the outcome was unknown for 21 (11%) cases. In addition, 27 patients (15%) were registered as alive and excreting at the latest specimen. However, for 20 of these patients, whose latest specimen was tested for poliovirus between 2011 and 2020, it is likely that they died or stopped shedding, or were lost to follow-up, but the outcome has not yet been reported to the registry.

#### 3.2.2. Risk of Poliomyelitis Among Patients with PID

The risk of poliomyelitis following WPV infection is likely higher among individuals with severe immunodeficiency than in immunocompetent individuals, and potentially they may have more severe disease. However, immunodeficient individuals probably died from poliomyelitis before their immunodeficiency was detected because PIDs were only recognized in the 1950s and the availability of treatment with gamma-globulin was limited to high income countries initially. In reports from three studies conducted in the UK in the early 1960s, eight of 154 (2%) patients with hypogammaglobulinemia developed poliomyelitis, above the expected rate of 0.1–1% among immunocompetent individuals [73].

Studies in the late 20th century showed that immunodeficient individuals had a much higher risk of paralytic poliomyelitis following exposure to OPV, and that paralysis could appear much later after exposure than expected in immunocompetent persons. The categorization as VAPP was considered in immunodeficient individuals even when vaccine exposure occurred well before the upper limits of 30 days (OPV recipients) to 75 days (contacts of recipients), criteria used for categorization as VAPP in immunocompetent individuals [7,13,57,62]. The identification of iVDPVs in the early 2000s showed that some VAPP cases in immunodeficient individuals were associated with iVDPVs infections. Since the early 2000s, some authors have reserved the term VAPP for paralytic poliomyelitis cases in immunodeficient individuals with Sabin-related virus isolated from patients’ specimens [68,74] vs. the term iVDPV case when iVDPVs are isolated; other authors have used the term VAPP for both situations [7,13,16,62,75,76].

Among VAPP cases reported during 1980–1991 to the US national poliomyelitis surveillance system, an immunodeficiency disorder was reported in 22 out of 98 cases (22%) and during 1990–2003, in 16 out of 59 cases (27%). The most common underlying immunological defects among the 22 patients reported during 1980–1991 were isolated B-cell deficiencies (73%), whereas combined B- and T-cell deficiencies accounted for 23%, and one patient (4%) was receiving immunosuppressive drugs [7,13]. Based upon estimates of the prevalence of PIDs, the authors projected that infants < 1 year of age with a PID had a risk of VAPP after receiving OPV > 3000 times higher than immunocompetent infants [7,13].

Among the 175 iVDPV case patients in the WHO Registry with a known clinical history, 106 (61%) were paralyzed (Table 2). Paralysis was more frequent in patients with B-cell deficiencies (50/60, 83%) than in patients with combined immunodeficiencies (19/54, 35%) or other categories of immune disorders (9/23, 39%) as reported in previous reviews [16,67,68]. For specific disorders, patients with agammaglobulinemia were most frequently paralyzed (22/23, 96%) followed by patients with hypogammaglobulinemia (10/13, 77%) and CVID (16/22, 73%) (Table 3). Although some authors have hypothesized that variable deficiencies in T-cell function may explain these differences in paralysis outcomes by type of immune disorder [16], it is also possible that longer duration of poliovirus infections of patients with B-cell deficiencies increases the risk of paralysis.

Paralytic poliomyelitis caused by Sabin-related vaccines remains extremely rare among patients with human immunodeficiency virus (HIV) infection, with only two cases reported to date, both likely caused by vaccine-like strains [77,78]. No paralysis related to iVDPV infection has been reported among patients with HIV infection to our knowledge.

#### 3.2.3. Potential Risk of Polio Outbreaks Initiated by Individuals with iVDPV Infection

Th re-introduction of poliovirus transmission into a polio-free country or region by an immunodeficient individual with iVDPV infection is theoretically possible. However, to date, we have very few instances of spread of iVDPV to close contacts and a few other instances when it is difficult to ascertain whether the immunodeficient individual initiated transmission or picked up a cVDPV from the community. In Spain, the same iVDPV isolated in July 2005 from a 14-month-old boy with major histocompatibility class II immunodeficiency (MHC-II) who had developed AFP was isolated in three of the seven family contacts tested. One of the contacts, considered immunocompetent, shed iVDPV for 216 days; no iVDPV was detected in sewage [79]. More recently, in India, genetically linked iVDPVs were isolated from a 9-month-old with SCID and his immunocompetent father in July–August 2022. Specimens collected from 114 additional household contacts and community members were negative. The child died within 3 months of diagnosis and the father presumably stopped shedding poliovirus [80]. In the United States, an iVDPV isolated in an unvaccinated Amish infant with SCID in September 2005 was genetically linked to VDPVs isolated from 8 out of 23 asymptomatic children tested from the same community in Minnesota, US. Phylogenetic analysis suggested that the initiating OPV dose had been given before the child’s birth [70]. Lastly, in the Philippines, a type 2 iVDPV detected in August 2019 in a 5-year-old boy with a complex immune disorder had the same genetic distance to parental Sabin-strain (7% divergence) as a cVDPV, causing an outbreak in the Philippines during September 2019–March 2020 [68,81].

The absence of evidence of iVDPV spread from immunodeficient individuals to the wider community has been attributed to: (1) high levels of herd immunity and good hygiene and sanitation in countries where children with PIDs are more likely to survive (high income countries); (2) low survival rate, for lack of treatment, of children with severe PIDs in developing countries where transmission is more likely to occur; and (3) the likelihood that iVDPV excretors may immunize their close contacts before they excrete highly diverged viruses, thus reducing their risk of initiating an outbreak [82]. However, with the decline in population mucosal immunity expected after global interruption of OPV use, the risk of spread of iVDPV will certainly increase with the potential of seeding outbreaks.

Severe PID with combined defects of B- and T-cells, like SCID, are infrequent and patients rarely survive beyond 1 year of age unless they respond to bone marrow transplant which also usually clears any enterovirus, including poliovirus, infection. On the other hand, B-cell immunodeficiencies are the most common PIDs and patients can survive for many years with uninterrupted immunoglobulin therapy and management of infections [36,37]. Considering that most of the chronic poliovirus infections have been detected in patients with CVID (8 out of 10 in our review), this PID group poses a higher risk of becoming a reservoir of neurovirulent poliovirus in the post-eradication era than other iVDPV-infected PID patients.

Estimations and modeling analysis of the risk that long-term iVDPV excretors pose in re-introducing live poliovirus transmission following WPV eradication is complex; very few PID patients present with prolonged poliovirus infection and there is uncertainty of the prevalence and survival rates of PID patients globally at present and in the future. The number of PID patients with iVDPV infection reported to the WHO Registry declined substantially in high-income countries following their shift to exclusive IPV use in the early 2000s but increased in middle income countries that continued to use OPV (Figure 2). In addition to improved detection and availability of therapy in more recent years, the higher number of iVDPV excretors detected in many countries in the Eastern Mediterranean region (e.g., Iran, Egypt, Tunisia) may be partially explained by the higher prevalence of consanguineous marriages compared to other countries that also use bOPV in routine immunization and have similar care opportunities for these patients. The estimated risk of long-term iVDPV excretors is low for PID patients residing in low-income countries, where the life expectancy of such patients is short [50,67,68,83].

Modeling analyses over time changed estimations of the global prevalence of prolonged iVDPV excretors from around 140 in 2006 [84] to about 6 chronic excretors (and 63 paralytic cases) during 2011–2018 [83]. These changes in estimates over time resulted from: (1) updated estimates of prevalence and survival of PIDs; (2) fewer prolonged excretors observed in cross-sectional studies and surveillance pilots conducted during 2000–2015 (~1%, Table 1) compared with earlier studies that followed VAPP case-cohorts during 1975–1997 (up to 10%) [85]; and (3) reductions in the number of iVDPV cases and changes in epidemiology after the shift to IPV-only immunization schedules in high-income and upper-middle income countries in the early 2000s, and after the global withdrawal of type 2 OPV in 2016 [14,83,86].

Following global cessation of OPV use for routine immunization and supplementary campaigns, the prevalence of chronic poliovirus excretors is expected to drop quickly, with very few excretors remaining >10 years after cessation in high- or middle-income countries [14,83,86]. New excretors may also appear if OPV is used in response to VDPV outbreaks post-cessation, as observed in Egypt where two PID patients with iVDPV infection were detected in 2022 following outbreak response campaigns delivering nOPV2 (source: WHO GPLN data). Despite the small number of prolonged excretors expected after OPV cessation, assuming that iVDPVs behave like homotypic WPV, modeling analyses conducted in 2015 and 2017 suggested that chronic iVDPV excretors residing in populations with medium-high infection risk (R0 > 6) could be responsible for outbreaks with large numbers of paralytic cases occurring several years after OPV cessation [14,86]. Efforts to identify and treat iVDPV excretors with antiviral drugs before OPV cessation would be cost-effective for the polio eradication initiative because it would reduce the likelihood and number of immunization campaigns required to stop post-cessation outbreaks. The net benefits of iVDPV surveillance and treatment would be considerably reduced, however, if antiviral drugs have low efficacy (i.e., 50% instead of 90%) or if iVDPV strains do not have the same transmissibility as homotypic WPV, which remains an important uncertainty [86].

### 3.3. Detection and Management of Patients with iVDPV Infection

#### 3.3.1. GPEI Efforts to Identify PID Individuals with iVDPV Infections

During 2008–2020, the GPEI supported surveillance research and pilot interventions that provided estimates of the prevalence of poliovirus excretion among PID patients in low, lower-, and upper-middle income countries. These pilot projects increased awareness among immunologists and physicians treating PIDs that likely contributed to increased case reporting during this period and also provided lessons for the subsequent roll-out of a surveillance platform [50,53,54,55,59,87,88,89,90]. First, a population-based platform that used AFP surveillance human resources to screen all patients admitted to a network hospital for potential PID, using broad clinical criteria for a definition of potential PID, was found to be extremely resource-intensive and not sustainable [91]. In a study conducted in Bangladesh during February 2011 through to January 2013, trained surveillance staff screened 96,000 children admitted to five hospitals for PID using the warning signs from the Jeffrey Model Foundation and found 53 patients who met the clinical definition of potential PID. Of these, 13 (24%) had confirmation of PID based upon a qualitative immunoglobulin assay; only one of 11 patients’ stool specimens tested was positive for poliovirus and became negative by 24 months later [87]. A similar project conducted in Egypt demonstrated a sharp decline in patients screened a few months after initiating the project unless frequent monitoring visits and training were implemented. Second, the involvement of physicians specialized in the diagnosis and treatment of PID patients was necessary to ensure coordination with poliovirus surveillance staff for sample collection, transportation, and testing, as well as reporting of results. Third, the availability of therapeutic options for clearing poliovirus infections was an important incentive for patients to accept initial and follow-up sampling and testing. Lastly, ownership by country public health was also essential to facilitate management of community investigations and to facilitate potential access to antiviral drugs.

Based upon the experience from these pilot projects, GPEI opted to support a sentinel-based surveillance platform in a number of low and lower-middle income countries considered at high risk. This platform relies on existing immunization program networks to report potential cases to WHO in countries where immunological diagnostic and treatment centers or experts are available. Parameters used to identify countries at risk for iVDPV emergences and transmission included: (1) predicted annual iVDPV country incidence based upon the high prevalence of consanguineous marriages, mortality among children below 5 years and total population; (2) population susceptibility, based upon coverage with three doses of OPV and one dose of IPV; and (3) risk of high poliovirus transmission, based upon mortality from diarrhea among children under 5 years old. The risk score was combined with information about the availability of care for PID patients and previous experience in detecting iVDPV infections. Countries with high scores included Pakistan, Iran, Egypt, Tunisia, China, Indonesia, India, Brazil, and Mexico.

During 2020–2024, GPEI guidelines and training modules for implementing poliovirus surveillance among patients with PID were developed and adapted by different WHO regional offices [92]. In addition, a data management system linking patient information with laboratory results was integrated within the WHO Polio information System (POLIS). Advocacy meetings and workshops were conducted with immunologists and surveillance focal points. As of June 2024, India and Pakistan have successfully transitioned from research-based surveillance projects to systematic surveillance with 21 and 98 sites, respectively. Egypt, Tunisia, and Iran are in transition and China continues a research-related surveillance project in three provinces, active since 2021. Program-based PID poliovirus surveillance was also initiated in Nigeria and Senegal in 2022 and in Cuba and Colombia in 2023.

However, implementation of poliovirus surveillance of PID patients has been hampered by several challenges in the last five years. Surveillance activities were markedly curtailed during the COVID-19 pandemic. Program and country perception of the risk of iVDPV carriers and urgency in establishing surveillance have declined as the GPEI and country programs have had to respond to outbreaks of type 2 cVDPV in >35 countries, endemic WPV1 circulation continues in Afghanistan and Pakistan, and country authorities are preoccupied with other urgent country health priorities. The GPEI has insufficient funding to sustain iVDPV surveillance in low-income countries with no immunology diagnostic capacity and there are no adequate mechanisms to transfer funds to middle-income countries transitioning from research projects. Lack of implementation of data sharing protocols and an absence of adequate data platform systems at the regional levels results in ad hoc reporting of iVDPV cases without systematic follow-up of patients to monitor the duration of iVDPV excretion and outcome of infection. Lastly, delays in the clinical development of an effective antiviral combination therapy and regulatory obstacles in some countries to the use of available antivirals are barriers to participation by parents of children with PID or by immunologists treating these patients.

All these challenges have reduced the sensitivity of poliovirus surveillance among PID individuals, which may be partly responsible for the observed reduction in the total iVDPV cases reported to the WHO Registry during 2019–2024. As an example, before submitting this manuscript, we found a case-report of a Pakistani patient, resident in Spain, with CVID and asymptomatic co-infection by iVDPV types 1 and 3 (divergence from parent Sabin strain 2.7% for type 1 and 1.5% for type 3). The case patient was identified in May 2019 but not reported to the WHO registry and our literature search missed it because it was reported as an “imported” VDPV case instead of an immunodeficiency-related VDPV case. The patient was still excreting iVDPV as of February 2020, after treatment with the antiviral pocapavir failed to interrupt infection [93].

#### 3.3.2. Management and Control Measures Recommended for PID Patients with iVDPV Infection

The GPEI guidelines for poliovirus surveillance in PID patients recommend testing for poliovirus infection those patients with low immunoglobulin levels for the age for whom transitory or secondary immunodeficiency (i.e., severe malnutrition, infection by HIV) have been excluded, and for whom a specialized physician confirms a diagnosis of a PID associated with risk of prolonged poliovirus excretion. Virologic testing is not recommended for patients with isolated immunoglobulin deficiencies (i.e., Ig A deficiency) [92].

Stool specimens should be sent to a global polio network laboratory for testing and, if necessary, genetic sequencing of poliovirus using the transportation logistics and GPLN resources available for AFP surveillance. Patients with negative specimens are recommended to have annual testing for potential new infection after exposure to vaccinees in essential immunization or campaigns [40,57,94]. For patients with specimens positive for poliovirus (vaccine-like strain or VDPV), a polio surveillance officer will conduct an investigation to assess the patient’s potential source of exposure and contact networks, polio vaccination status and potential infection among close contacts, and risk of transmission in the community (i.e., vaccination coverage, population density, sanitation). Collection of stool samples from household contacts and from a sample of young children in the community are recommended to rule out circulation.

Management of the PID patient with poliovirus infection includes counseling and education for the patient and family on hand and toilet hygiene to prevent transmission to contacts, vaccination of close contacts with IPV if required, and vaccination of health staff and adherence to standard precautions for infection control in health care facilities. Monthly stool specimen collections are recommended to monitor clearance of vaccine-like poliovirus or evolution to iVDPV.

Treatment with regular immunoglobulin therapy or bone marrow transplant, in addition to management of infections, depends on the type of PID and the country level of care. Treatment with antivirals is to be considered in PID individuals excreting WPV or iVDPV in any stool specimen and in patients excreting vaccine-like virus for longer than 2 months. As of April 2023, a total of 12 patients had received Pocapavir (a capsid inhibitor), with successful clearance of the iVDPV infection observed in six patients (50%), and the development of resistant poliovirus strains in the remaining patients. A combination of Pocapavir with Imocitrelvir, a protease inhibitor, is still under clinical development. In addition to the emergence of viral resistance with a single agent, a major challenge for the provision of antiviral drugs for more patients with iVDPV infection has been the absence of regulatory mechanisms that allow their use as an investigational drug or for compassionate use in a number of countries, like Egypt or India.

## 4. Conclusions and Future Directions

Prolonged poliovirus infection following exposure to OPV in patients with certain primary immunodeficiencies is associated with high risk of paralysis for the individual and an uncertain risk of re-introduction of poliovirus in a polio-free world. Surveillance for poliovirus infection among PID patients and prompt treatment with effective antiviral drugs, when available, is crucial to reduce the risk posed by patients with iVDPV infection for their own health and for the global eradication endgame. In this updated analysis of the WHO Registry of patients with iVDPV infection, we observed a sharp reduction in the number of type 2 iVDPV cases reported during 2020–2024, as expected after the global switch from tOPV to bOPV that removed OPV type 2 from use. However, because reported cases with iVDPV types 1 and 3 also increased in this period, the actual decline in total iVDPV cases was small. This analysis also confirmed the trend of a higher proportion of cases reported from middle income countries than from high income countries, because OPV remains in use in the former and where there is capacity for PID diagnosis and treatment. The higher number of cases reported from specific countries such as Egypt or Iran are explained by the higher prevalence of certain PIDs associated with consanguineous marriages, and their high population/number of births.

The GPEI supports establishing sensitive surveillance for the detection of iVDPV infection among patients with PIDs in sentinel sites in countries considered at a higher risk for the emergence and potential transmission of iVDPVs. However, following successful pilots in certain countries, efforts to roll out a program-based surveillance for poliovirus infections in PIDs have been challenged by competing health priorities in high-risk countries and within the GPEI and by the lack of availability of highly effective antiviral drugs. The development of resistance to a single drug and bureaucratic challenges to using antiviral agents for investigational use or under compassionate use limits timely access for a number of patients in some countries; the small number of patients available for testing new antivirals or combinations further slows their clinical development.

Despite the challenges, the GPEI plans to continue advocating and supporting the implementation of surveillance for poliovirus infections among patients with PID and ultimate access to antiviral treatment. In collaboration with PATH, a new study is comparing, in Pakistan, the accuracy of a rapid screening tests for the detection of patients with low IgG levels, which may facilitate the implementation of surveillance in low-income countries without high diagnostic capacity. A combination therapy of Pocapavir, a capsid inhibitor, with Imocitrelvir, a protease inhibitor, less likely to induce resistance, has completed Phase 1 clinical trials and is expected to go into further clinical trials in India in 2025 (further information in a separate article in this supplement).

## Figures and Tables

**Table 1:** Prevalence of poliovirus shedding among patients with primary immunodeficiency disorders (PID) in cross-sectional studies.

Country	Patients with PIDs Studied	Poliovirus Excretors		iVDPV Excretors		Years of Study	Reference
Bangladesh	13	1	8%	0	0%	2008–2013	[54]
Brazil	95	3	3%	0	0%	2001–2002	[45]
China	167	3	2%	0	0%	2008–2013	[54]
China	63	0	0%	0	0%	2014–2015	[50]
Colombia	25	0	0%	0	0%	2014–2015	[50]
Egypt	15	2	13%	0	0%	2006–2009	[59]
Egypt	104	12	12%	6	6%	2011–2014	[51]
India	23	0	0%	0	0%	2014–2015	[50]
India	42	3	7%	1	2%	2014–2017	[55]
India	154	4	3%	1	1%	2019	[56]
Iran	43	1	2%	0	0%	2008–2013	[54]
Iran	102	4	4%	3	3%	2014–2015	[50]
Israel	24	0	0%	0	0%	2014–2015	[50]
Italy	38	0	0%	0	0%	2001–2002	[52]
Japan	9	0	0%	0	0%	2014–2015	[50]
Mexico	33	1	3%	0	0%	2001–2002	[45]
Mexico	20	0	0%	0	0%	2014–2015	[50]
Poland	0	0	0%	0	0%	2014–2015	[50]
Philippines	70	1	1%	0	0%	2008–2013	[54]
Sri Lanka	51	5	10%	2	4%	2008–2013	[54,60]
Russia	136	0	0%	0	0%	2008–2013	[54]
Russia	83	2	2%	0	0%	2014–2015	[50]
Tunisia	16	4	25%	0	0%	1996–1997	[57]
Tunisia	82	6	7%	0	0%	2008–2013	[54,58]
Tunisia	40	3	8%	1	3%	2014–2015	[50]
Turkey	172	4	2%	1	1%	2014–2015	[50]
United Kingdom	125	0	0%	0	0%	2001–2002	[45]
United States	94	0	0%	0	0%	2001–2002	[45]
**Total**	**1717**	**59**	**3%**	**15**	**1%**		

**Table 2:** Characteristics of 184 case patients with immunodeficiency-associated vaccine-derived poliovirus (iVDPV) reported during 1962–2024 (data as of July 2024).

Variable	Categories	iVDPV Cases, n (%)
**Gender**	Male	103 (56)
	Female	67 (36)
	Missing	14 (8)
**Age at onset or first positive specimen**	<1 year	83 (45)
	1 to <5 years	64 (34)
	5 to <10 years	11 (6)
	10 to <20 years	7 (4)
	20 to <30 years	3 (2)
	≥30 years	2 (1)
	Missing	14 (8)
**Paralysis**	Yes	106 (58)
	No	69 (38)
	Unknown	9 (5)
**Country of residence World Bank income classification**	High income	38 (21)
	Upper-middle income	77 (42)
	Lower-middle income	68 (37)
	Low income	1 (1)
**WHO region of residence**	African Region	14 (8)
	Eastern Mediterranean Region	88 (48)
	European Region	24 (13)
	Region of the Americas	18 (10)
	South-East Asian Region	20 (11)
	Western Pacific Region	20 (11)
**B-cell immunodeficiencies**	CVID	23 (12)
	AGG	23 (12)
	HGG	13 (7)
	Other B-cell immunodeficiencies	2 (1)
**Combined B- and T-cell immunodeficiencies**	SCID	45 (24)
	MHC class II deficiency	19 (10)
	Other combined immunodeficiencies	9 (5)
**Other immune disorders**	Other disorders	4 (2)
	Unknown	46 (25)
**Poliovirus serotype** **^a^**	1	42 (22)
	2	97 (53)
	3	50 (27)
**Outcome status**	Dead	74 (40)
	Alive (stopped excreting)	62 (34)
	Alive (excreting at last specimen)	27 (15)
	Unknown	21 (11)

^a^ Five patients had co-infection and are counted twice: three patients were co-infected with types 1 and 2, one patient with types 2 and 3, and one patient with types 1 and 3. AGG—agammaglobulinemia, CVID—common variable immunodeficiency disorder, HGG—hypogammaglobulinemia, MHC II—Major histocompatibility complex type 2, SCID—severe combined immunodeficiency disorder.

**Table 3:** Characteristics of case patients with immunodeficiency-associated vaccine-derived poliovirus (iVDPV) reported during 1962–2024 by type of immunodeficiency disorder, clinical outcome, and virus characteristics (data as of July 2024).

Immunodeficiency Disorder	Patients n (%)	Paralysis n (%)	Dead n (%)	Alive (Excreting at Last Specimen) n (%)	Number of Nucleotides at Time of Detection Median (Range)	Length of Excretion in Years Median (Range)	Type 1 n (%)	Type 2 n (%)	Type 3 n (%)
B-cell immunodeficiencies									
AGG	23 (13.2)	22 (96%)	4 (17%)	1 (4%)	1.5 (0.5–3.5)	0.3 (0.05–4.92)	5 (22%)	14 (61%)	4 (17%)
CVID	22 (12.6)	16 (73%)	8 (36%)	1 (5%)	2.2 (0.7–12.3)	0.625 (0.04–26.42)	6 (27%)	13 (59%)	4 (18%)
HGG	13 (7.5)	10 (77%)	8 (62%)	0 (0%)	1.55 (0.6–2.2)	0.485 (0.01–1.83)	2 (15%)	9 (69%)	2 (15%)
Other antibody disorders	2 (1.1)	2 (100%)	0 (0%)	1 (50%)	1.1 (0.6–1.6)	0.45 (0.45–0.45)	1 (50%)	1 (50%)	0 (0%)
Combined B- and T-cell immunodeficiencies									
SCID	45 (25.9)	16 (36%)	30 (67%)	3 (7%)	1.4 (0.66–4.5)	0.2 (0.02–2.13)	11 (24%)	28 (62%)	9 (20%)
MHC class II deficiency	19 (10.9)	7 (37%)	8 (42%)	3 (16%)	1.6 (0.67–4)	0.405 (0.09–1.74)	3 (16%)	9 (47%)	7 (37%)
Other combined immunodeficiencies	9 (5.2)	3 (33%)	3 (33%)	0 (0%)	1.7 (1–2.2)	0.205 (0.05–0.46)	3 (33%)	3 (33%)	3 (33%)
Other immunodeficiency disorders									
Other disorders	4 (2.3)	2 (50%)	1 (25%)	0 (0%)	1 (0.9–1.6)	0.68 (0.07–1.29)	1 (25%)	3 (75%)	0 (0%)
Unknown	47 (27)	28 (60%)	11 (23%)	21 (45%)	1.35 (0.67–7)	0.505 (0.01–3.37)	10 (21%)	17 (36%)	21 (45%)

AGG—Agammaglobulinemia, HGG—Hypogammaglobulinemia, CVID—common variable immunodeficiency disorder, MHC—Major histocompatibility complex, SCID—severe combined immunodeficiency disorder.

## Data Availability

No new data were created for this review; data used for this review are available in the published peer preview literature and Supplementary Materials.

## Supplementary Materials

The following supporting information can be downloaded at: https://www.mdpi.com/article/10.3390/pathogens13121128/s1, Table S1: Characteristics of iVDPV cases in the WHO Registry as of June 2023; Figure S1: VP1 divergence of isolated iVPDV from parental Sabin strains, by serotype and category of immunodeficiency [95,96,97,98,99,100,101,102,103,104,105,106,107,108,109,110,111,112,113,114,115,116,117,118,119,120,121,122,123,124,125,126,127].

## References

[1] Global Polio Eradication Initiative List of Wild Poliovirus by Country and Year. http://polioeradication.org/polio-today/polio-now/wild-poliovirus-list.

[2] Estivariz C.F., Burns C.C., Macklin G.R., Orenstein W.A., Offit P.A., Edwards K.M., Plotkin S.A. (2023). Poliovirus Vaccine—Live. Vaccines.

[3] Sutter R.W., Patriarca P.A., Kurstak E. (1993). Inactivated and live, attenuated poliovirus vaccines: Mucosal immunity. Measles and Poliomyelitis.

[4] Agol V.I. (2006). Vaccine-derived polioviruses. Biologicals.

[5] Burns C.C., Diop O.M., Sutter R.W., Kew O.M. (2014). Vaccine-derived polioviruses. J. Infect. Dis..

[6] Kew O.M., Wright P.F., Agol V.I., Delpeyroux F., Shimizu H., Nathanson N., Pallansch M.A. (2004). Circulating vaccine-derived polioviruses: Current state of knowledge. Bull. World Health Organ..

[7] Alexander L.N., Seward J.F., Santibanez T.A., Pallansch M.A., Kew O.M., Prevots D.R., Strebel P.M., Cono J., Wharton M., Orenstein W.A. (2004). Vaccine policy changes and epidemiology of poliomyelitis in the United States. JAMA.

[8] Platt L.R., Estivariz C.F., Sutter R.W. (2014). Vaccine-associated paralytic poliomyelitis: A review of the epidemiology and estimation of the global burden. J. Infect. Dis..

[9] Estivariz C.F., Watkins M.A., Handoko D., Rusipah R., Deshpande J., Rana B.J., Irawan E., Widhiastuti D., Pallansch M.A., Thapa A. (2008). A large vaccine-derived poliovirus outbreak on Madura Island–Indonesia, 2005. J. Infect. Dis..

[10] Jenkins H.E., Aylward R.B., Gasasira A., Donnelly C.A., Abanida E.A., Koleosho-Adelekan T., Grassly N.C. (2008). Effectiveness of immunization against paralytic poliomyelitis in Nigeria. N. Engl. J. Med..

[11] Global Polio Eradication Initiative Responding to a Poliovirus Event or Outbreak Part 2: Protocol for Poliovirus Type 2 (V 2.4). Geneva: World Health Organization..

[12] Global Polio Eradication Initiative Polio Eradication & Endgame Strategic Plan 2013–2018. WHO/POLIO/13.02. Geneva, Switzerland..

[13] Sutter R.W., Prevots D.R. (1994). Vaccine-Associated Paralytic Poliomyelitis Among Immunodeficient Persons. Infect. Med..

[14] Duintjer Tebbens R.J., Pallansch M.A., Thompson K.M. (2015). Modeling the prevalence of immunodeficiency-associated long-term vaccine-derived poliovirus excretors and the potential benefits of antiviral drugs. BMC Infect. Dis..

[15] Macklin G., Liao Y., Takane M., Dooling K., Gilmour S., Mach O., Kew O.M., Sutter R.W., The iVDPV Working Group (2017). Prolonged Excretion of Poliovirus among Individuals with Primary Immunodeficiency Disorder: An Analysis of the World Health Organization Registry. Front. Immunol..

[16] Shaghaghi M., Soleyman-Jahi S., Abolhassani H., Yazdani R., Azizi G., Rezaei N., Barbouche M.R., McKinlay M.A., Aghamohammadi A. (2018). New insights into physiopathology of immunodeficiency-associated vaccine-derived poliovirus infection; systematic review of over 5 decades of data. Vaccine.

[17] World Health Organization (2004). Department of Immunization, Vaccines and Biologicals. Polio laboratory Manual.

[18] Jorba J., Campagnoli R., De L., Kew O. (2008). Calibration of multiple poliovirus molecular clocks covering an extended evolutionary range. J. Virol..

[19] Hammon W.M., Coriell L.L., Ludwig E.H., Mc A.R., Greene A.E., Sather G.E., Wehrle P.F. (1954). Evaluation of Red Cross gamma globulin as a prophylactic agent for poliomyelitis. 5. Reanalysis of results based on laboratory-confirmed cases. J. Am. Med. Assoc..

[20] Wahid R., Cannon M.J., Chow M. (2005). Virus-specific CD4+ and CD8+ cytotoxic T-cell responses and long-term T-cell memory in individuals vaccinated against polio. J. Virol..

[21] Ghendon Y., Sanakoyeva I.I. (1961). Comparison of the resistance of the intestinal tract ot poliomyelitis virus (Sabin’s strains) in persons after naturally and experimentally acquired immunity. Acta Virol..

[22] Alexander J.P., Gary H.E., Pallansch M.A. (1997). Duration of poliovirus excretion and its implications for acute flaccid paralysis surveillance: A review of the literature. J. Infect. Dis..

[23] Onorato I.M., Modlin J.F., McBean A.M., Thoms M.L., Losonsky G.A., Bernier R.H. (1991). Mucosal immunity induced by enhanced-potency inactivated and oral polio vaccines. J. Infect. Dis..

[24] Ogra P.L. (1984). Mucosal immune response to poliovirus vaccines in childhood. Rev. Infect. Dis..

[25] Valtanen S., Roivainen M., Piirainen L., Stenvik M., Hovi T. (2000). Poliovirus-Specific Intestinal Antibody Responses Coincide with Decline of Poliovirus Excretion. J. Infect. Dis..

[26] Ogra P.L., Fishaut M., Gallagher M.R. (1980). Viral vaccination via the mucosal routes. Rev. Infect. Dis..

[27] Ghendon Y., Robertson S.E. (1994). Interrupting the transmission of wild polioviruses with vaccines: Immunological considerations. Bull. World Health Organ..

[28] Abbink F., Buisman A.M., Doornbos G., Woldman J., Kimman T.G., Conyn-van Spaendonck M.A. (2005). Poliovirus-specific memory immunity in seronegative elderly people does not protect against virus excretion. J. Infect. Dis..

[29] Faden H., Duffy L., Sun M., Shuff C. (1993). Long-term immunity to poliovirus in children immunized with live attenuated and enhanced-potency inactivated trivalent poliovirus vaccines. J. Infect. Dis..

[30] Nathanson N., Kew O.M. (2010). From emergence to eradication: The epidemiology of poliomyelitis deconstructed. Am. J. Epidemiol..

[31] Macadam A.J., Pollard S.R., Ferguson G., Skuce R., Wood D., Almond J.W., Minor P.D. (1993). Genetic basis of attenuation of the Sabin type 2 vaccine strain of poliovirus in primates. Virology.

[32] Georgescu M.M., Balanant J., Macadam A., Otelea D., Combiescu M., Combiescu A.A., Crainic R., Delpeyroux F. (1997). Evolution of the Sabin type 1 poliovirus in humans: Characterization of strains isolated from patients with vaccine-associated paralytic poliomyelitis. J. Virol..

[33] Global Polio Eradication Initiative Classification and Reporting of Vaccine-Derived Polioviruses (VDPVs). http://polioeradication.org/wp-content/uploads/2016/09/Reporting-and-Classification-of-VDPVs_Aug2016_EN.pdf.

[34] Tangye S.G., Al-Herz W., Bousfiha A., Cunningham-Rundles C., Franco J.L., Holland S.M., Klein C., Morio T., Oksenhendler E., Picard C. (2022). Human Inborn Errors of Immunity: 2022 Update on the Classification from the International Union of Immunological Societies Expert Committee. J. Clin. Immunol..

[35] Abolhassani H., Azizi G., Sharifi L., Yazdani R., Mohsenzadegan M., Delavari S., Sohani M., Shirmast P., Chavoshzadeh Z., Mahdaviani S.A. (2020). Global systematic review of primary immunodeficiency registries. Expert Rev. Clin. Immunol..

[36] Yong P.F.K., Grimbacher B., Thaventhiran J.E.D. (2011). “A Rose is a Rose is a Rose,” but CVID is Not CVID. Adv. Immunol..

[37] Rosen F.S., Cooper M.D., Wedgwood R.J. (1995). The primary immunodeficiencies. N. Eng. J. Med..

[38] MacCallum F.O. (1971). Hypogammaglobulinaemia in the United Kingdom. VII. The role of humoral antibodies in protection against and recovery from bacterial and virus infections in hypogammaglobulinaemia. Spec. Rep. Ser. Med. Res. Counc..

[39] Martín J. (2006). Vaccine-derived poliovirus from long term excretors and the end game of polio eradication. Biologicals.

[40] Weil M., Shulman L.M., Heiman S., Stauber T., Alfandari J., Weiss L., Silberstein I., Indenbaum V., Mendelson E., Sofer D. (2016). Prolonged excretion of type-2 poliovirus from a primary immune deficient patient during the transition to a type-2 poliovirus-free world, Israel, 2016. Eurosurveillance.

[41] Odoom J.K., Yunus Z., Dunn G., Minor P.D., Martin J. (2008). Changes in population dynamics during long-term evolution of sabin type 1 poliovirus in an immunodeficient patient. J. Virol..

[42] Yang C.F., Chen H.Y., Jorba J., Sun H.C., Yang S.J., Lee H.C., Huang Y.C., Lin T.Y., Chen P.J., Shimizu H. (2005). Intratypic recombination among lineages of type 1 vaccine-derived poliovirus emerging during chronic infection of an immunodeficient patient. J. Virol..

[43] Kew O.M., Sutter R.W., Nottay B.K., McDonough M.J., Prevots D.R., Quick L., Pallansch M.A. (1998). Prolonged replication of a type 1 vaccine-derived poliovirus in an immunodeficient patient. J. Clin. Microbiol..

[44] Savilahti E., Klemola T., Carlsson B., Mellander L., Stenvik M., Hovi T. (1988). Inadequacy of mucosal IgM antibodies in selective IgA deficiency: Excretion of attenuated polio viruses is prolonged. J. Clin. Immunol..

[45] Halsey N.A., Pinto J., Espinosa-Rosales F., Faure-Fontenla M.A., da Silva E., Khan A.J., Webster A.D., Minor P., Dunn G., Asturias E. (2004). Search for poliovirus carriers among people with primary immune deficiency diseases in the United States, Mexico, Brazil, and the United Kingdom. Bull. World Health Organ..

[46] Asturias E.J., Grazioso C.F., Luna-Fineman S., Torres O., Halsey N.A. (2006). Poliovirus excretion in Guatemalan adults and children with HIV infection and children with cancer. Biologicals.

[47] Hennessey K.A., Lago H., Diomande F., Akoua-Koffi C., Caceres V.M., Pallansch M., Kew O., Nolan M., Zuber P. (2005). Poliovirus vaccine shedding among persons with HIV in Abidjan, Cote d’Ivoire. J. Infect. Dis..

[48] Pavlov D.N., Van Zyl W.B., Kruger M., Blignaut L., Grabow W.O., Ehlers M.M. (2006). Poliovirus vaccine strains detected in stool specimens of immunodeficient children in South Africa. Diagn. Microbiol. Infect. Dis..

[49] Ryder R.W., Oxtoby M.J., Mvula M., Batter V., Baende E., Nsa W., Davachi F., Hassig S., Onorato I., Deforest A. (1993). Safety and immunogenicity of bacille Calmette-Guerin, diphtheria-tetanus-pertussis, and oral polio vaccines in newborn children in Zaire infected with human immunodeficiency virus type 1. J. Pediatr..

[50] Aghamohammadi A., Abolhassani H., Kutukculer N., Wassilak S.G., Pallansch M.A., Kluglein S., Quinn J., Sutter R.W., Wang X., Sanal O. (2017). Patients with Primary Immunodeficiencies Are a Reservoir of Poliovirus and a Risk to Polio Eradication. Front. Immunol..

[51] El-Sayed Z.A., Mach O., Hossny E., Galal N.M., El-Sawy I., ElMarsafy A., Reda S.M., Moussa I., Sibak M.A., Bassiouni L. (2016). Poliovirus excretion among persons with Primary Immune Deficiency disorders: Summary of data from enhanced poliovirus surveillance in Egypt, 2011–2014. J. Vaccines Vaccin..

[52] Fiore L., Novello F., Simeoni P., Amato C., Vellucci L., De Stefano D., Grandolfo M.E., Luzzi I. (1999). Surveillance of acute flaccid paralysis in Italy: 1996–1997. AFP Study Group. Acute flaccid paralysis. Eur. J. Epidemiol..

[53] Galal N.M., Meshaal S., ElHawary R., Nasr E., Bassiouni L., Ashghar H., Farag N.H., Mach O., Burns C., Iber J. (2018). Poliovirus excretion following vaccination with live poliovirus vaccine in patients with primary immunodeficiency disorders: Clinicians’ perspectives in the endgame plan for polio eradication. BMC Res. Notes.

[54] Li L., Ivanova O., Driss N., Tiongco-Recto M., da Silva R., Shahmahmoodi S., Sazzad H.M., Mach O., Kahn A.L., Sutter R.W. (2014). Poliovirus excretion among persons with primary immune deficiency disorders: Summary of a seven-country study series. J. Infect. Dis..

[55] Mohanty M.C., Madkaikar M.R., Desai M., Taur P., Nalavade U.P., Sharma D.K., Gupta M., Dalvi A., Shabrish S., Kulkarni M. (2017). Poliovirus Excretion in Children with Primary Immunodeficiency Disorders, India. Emerg. Infect. Dis..

[56] Mohanty M.C., Desai M., Mohammad A., Aggarwal A., Govindaraj G., Bhattad S., Lashkari H.P., Rajasekhar L., Verma H., Kumar A. (2023). Assessment of Enterovirus Excretion and Identification of VDPVs in Patients with Primary Immunodeficiency in India: Outcome of ICMR-WHO Collaborative Study Phase-I. Vaccines.

[57] Triki H., Barbouche M.B., Bahri O., Bejaoui M., Dellagi K. (2003). Community-acquired poliovirus infection in children with primary immunodeficiencies in Tunisia. J. Clin. Microbiol..

[58] Driss N., Ben-Mustapha I., Mellouli F., Ben Yahia A., Touzi H., Bejaoui M., Ben Ghorbel M., Triki H., Barbouche M.R. (2012). High susceptibility for enterovirus infection and virus excretion features in Tunisian patients with primary immunodeficiencies. Clin. Vaccine Immunol..

[59] Galal N.M., Bassiouny L., Nasr E., Abdelmeguid N. (2012). Isolation of poliovirus shedding following vaccination in children with antibody deficiency disorders. J. Infect. Dev. Ctries..

[60] de Silva R., Gunasena S., Ratnayake D., Wickremesinghe G.D., Kumarasiri C.D., Pushpakumara B.A., Deshpande J., Kahn A.L., Sutter R.W. (2012). Prevalence of prolonged and chronic poliovirus excretion among persons with primary immune deficiency disorders in Sri Lanka. Vaccine.

[61] Khetsuriani N., Prevots D.R., Quick L., Elder M.E., Pallansch M., Kew O., Sutter R.W. (2003). Persistence of vaccine-derived polioviruses among immunodeficient persons with vaccine-associated paralytic poliomyelitis. J. Infect. Dis..

[62] Centers for Disease Control and Prevention (1997). Prolonged poliovirus excretion in an immunodeficient person with vaccine-associated paralytic poliomyelitis. MMWR Morb. Mortal. Wkly. Rep..

[63] Centers for Disease Control and Prevention (2006). Update on vaccine-derived polioviruses. MMWR Morb. Mortal. Wkly. Rep..

[64] Bellmunt A., May G., Zell R., Pring-Akerblom P., Verhagen W., Heim A. (1999). Evolution of poliovirus type I during 5.5 years of prolonged enteral replication in an immunodeficient patient. Virology.

[65] MacLennan C.A., Huissoon A.P., Kumararatne D.S. (2011). Vaccine-derived poliomyelitis 12 years after infection. N. Engl. J. Med..

[66] Bermingham W.H., Canning B., Wilton T., Kidd M., Klapsa D., Majumdar M., Sooriyakumar K., Martin J., Huissoon A.P. (2023). Case report: Clearance of longstanding, immune-deficiency-associated, vaccine-derived polio virus infection following remdesivir therapy for chronic SARS-CoV-2 infection. Front. Immunol..

[67] Guo J., Bolivar-Wagers S., Srinivas N., Holubar M., Maldonado Y. (2015). Immunodeficiency-related vaccine-derived poliovirus (iVDPV) cases: A systematic review and implications for polio eradication. Vaccine.

[68] Macklin G., Diop O.M., Humayun A., Shahmahmoodi S., El-Sayed Z.A., Triki H., Rey G., Avagyan T., Grabovac V., Jorba J. (2020). Update on Immunodeficiency-Associated Vaccine-Derived Polioviruses—Worldwide, July 2018–December 2019. MMWR Morb. Mortal. Wkly. Rep..

[69] Mohanty M.C., Madkaikar M.R., Desai M., Aluri J., Varose S.Y., Taur P., Sharma D.K., Nalavade U.P., Rane S.V., Gupta M. (2019). Natural Clearance of Prolonged VDPV Infection in a Child With Primary Immunodeficiency Disorder. Front. Immunol..

[70] Alexander J.P., Ehresmann K., Seward J., Wax G., Harriman K., Fuller S., Cebelinski E.A., Chen Q., Jorba J., Kew O.M. (2009). Transmission of imported vaccine-derived poliovirus in an undervaccinated community in Minnesota. J. Infect. Dis..

[71] Singanayagam A., Klapsa D., Burton-Fanning S., Hand J., Wilton T., Stephens L., Mate R., Shillitoe B., Celma C., Slatter M. (2023). Asymptomatic immunodeficiency-associated vaccine-derived poliovirus infections in two UK children. Nat. Commun..

[72] Bandyopadhyay A.S., Zipursky S. (2023). A novel tool to eradicate an ancient scourge: The novel oral polio vaccine type 2 story. Lancet Infect. Dis..

[73] Wyatt H.V. (1973). Poliomyelitis in hypogammaglobulinemics. J. Infect. Dis..

[74] Jorba J., Diop O.M., Iber J., Sutter R.W., Wassilak S.G., Burns C.C. (2016). Update on Vaccine-Derived Polioviruses—Worldwide, January 2015–May 2016. MMWR Morb. Mortal. Wkly. Rep..

[75] Shaghaghi M., Parvaneh N., Ostad-Rahimi P., Fathi S.M., Shahmahmoodi S., Abolhassani H., Aghamohammadi A. (2014). Combined immunodeficiency presenting with vaccine-associated paralytic poliomyelitis: A case report and narrative review of literature. Immunol. Investig..

[76] Shahmahmoodi S., Mamishi S., Aghamohammadi A., Aghazadeh N., Tabatabaie H., Gooya M.M., Zahraei S.M., Mousavi T., Yousefi M., Farrokhi K. (2010). Vaccine-associated paralytic poliomyelitis in immunodeficient children, Iran, 1995–2008. Emerg. Infect. Dis..

[77] Ion-Nedelcu N., Dobrescu A., Strebel P.M., Sutter R.W. (1994). Vaccine-associated paralytic poliomyelitis and HIV infection. Lancet.

[78] Chitsike I., van Furth R. (1999). Paralytic poliomyelitis associated with live oral poliomyelitis vaccine in child with HIV infection in Zimbabwe: Case report. BMJ.

[79] Avellon A., Cabrerizo M., de Miguel T., Perez-Brena P., Tenorio A., Perez J.L., de Aragon M.V., Trallero G. (2008). Paralysis case and contact spread of recombinant vaccine-derived poliovirus, Spain. Emerg. Infect. Dis..

[80] Mohanty M.C., Govindaraj G., Ahmad M., Varose S.Y., Tatkare M., Shete A., Yadav S., Joshi Y., Yadav P., Sharma D. (2024). Immunodeficiency-Related Vaccine-Derived Poliovirus (iVDPV) Excretion in an Infant with Severe Combined Immune Deficiency with Spillover to a Parent. Vaccines.

[81] Snider C.J., Boualam L., Tallis G., Takashima Y., Abeyasinghe R., Lo Y.R., Grabovac V., Avagyan T., Aslam S.K., Eltayeb A.O. (2023). Concurrent outbreaks of circulating vaccine-derived poliovirus types 1 and 2 affecting the Republic of the Philippines and Malaysia, 2019–2021. Vaccine.

[82] Dowdle W.R., De Gourville E., Kew O.M., Pallansch M.A., Wood D.J. (2003). Polio eradication: The OPV paradox. Rev. Med. Virol..

[83] Kalkowska D.A., Pallansch M.A., Thompson K.M. (2019). Updated modelling of the prevalence of immunodeficiency-associated long-term vaccine-derived poliovirus (iVDPV) excreters. Epidemiol. Infect..

[84] Tebbens R.J., Sangrujee N., Thompson K.M. (2006). The costs of future polio risk management policies. Risk Anal..

[85] Khetsuriani N., Helfand R., Pallansch M., Kew O., Fowlkes A., Oberste M.S., Tukei P., Muli J., Makokha E., Gary H. (2009). Limited duration of vaccine poliovirus and other enterovirus excretion among human immunodeficiency virus infected children in Kenya. BMC Infect. Dis..

[86] Duintjer Tebbens R.J., Thompson K.M. (2017). Comprehensive screening for immunodeficiency-associated vaccine-derived poliovirus: An essential oral poliovirus vaccine cessation risk management strategy. Epidemiol. Infect..

[87] Sazzad H.M., Rainey J.J., Kahn A.L., Mach O., Liyanage J.B., Alam A.N., Kawser C.A., Hossain A., Sutter R., Luby S.P. (2014). Screening for long-term poliovirus excretion among children with primary immunodeficiency disorders: Preparation for the polio posteradication era in Bangladesh. J. Infect. Dis..

[88] Shaghaghi M., Shahmahmoodi S., Abolhassani H., Soleyman-Jahi S., Parvaneh L., Mahmoudi S., Chavoshzadeh Z., Yazdani R., Zahraei S.M., Ebrahimi M. (2016). Vaccine-Derived Polioviruses and Children with Primary Immunodeficiency, Iran, 1995–2014. Emerg. Infect. Dis..

[89] Shaghaghi M., Shahmahmoodi S., Nili A., Abolhassani H., Madani S.P., Nejati A., Yousefi M., Kandelousi Y.M., Irannejad M., Shaghaghi S. (2019). Vaccine-Derived Poliovirus Infection among Patients with Primary Immunodeficiency and Effect of Patient Screening on Disease Outcomes, Iran. Emerg. Infect. Dis..

[90] Yao N., Liu Y., Xu J.W., Wang Q., Yin Z.D., Wen N., Yang H., Rodewald L.E., Zhang Z.Y. (2022). Detection of a Highly Divergent Type 3 Vaccine-Derived Poliovirus in a Child with a Severe Primary Immunodeficiency Disorder—Chongqing, China, 2022. MMWR Morb. Mortal. Wkly. Rep..

[91] World Health Organization (2016). Meeting of the Strategic Advisory Group of Experts on immunization, October 2016—Conclusions and recommendation. Wkly. Epidemiol. Rec..

[92] Global Polio Eradication Initiative (2022). Guidelines for Implementing Polio Surveillance Among Patients with Primary Immunodeficiency Disorders (PIDs).

[93] Álamo-Junquera D., Politi J., Simón P., Dieli-Crimi R., Borrell R.P., Colobran R., Martínez-Gallo M., Campins M., Antón A., Esperalba J. (2021). Coordinated Response to Imported Vaccine-Derived Poliovirus Infection, Barcelona, Spain, 2019–2020. Emerg. Infect. Dis..

[94] DeVries A.S., Harper J., Murray A., Lexau C., Bahta L., Christensen J., Cebelinski E., Fuller S., Kline S., Wallace G.S. (2011). Vaccine-derived poliomyelitis 12 years after infection in Minnesota. N. Engl. J. Med..

[95] Kew O.M., Sutter R.W., de Gourville E.M., Dowdle W.R., Pallansch M.A. (2005). Vaccine-derived polioviruses and the endgame strategy for global polio eradication. Ann. Rev. Microbiol..

[96] Minor P. (2009). Vaccine-derived poliovirus (VDPV): Impact on poliomyelitis eradication. Vaccine.

[97] Labadie K., Pelletier I., Saulnier A., Martin J., Colbere-Garapin F. (2004). Poliovirus mutants excreted by a chronically infected hypogammaglobulinemic patient establish persistent infections in human intestinal cells. Virology.

[98] Yoneyama T., Hagiwara A., Hara M., Shimojo H. (1982). Alteration in oligonucleotide fingerprint patterns of the viral genome in poliovirus type 2 isolated from paralytic patients. Infect. Immun..

[99] Hara M., Saito Y., Komatsu T., Kodama H., Abo W., Chiba S., Nakao T. (1981). Antigenic analysis of polioviruses isolated from a child with agammaglobulinemia and paralytic poliomyelitis after Sabin vaccine administration. Microbiol. Immunol..

[100] Abo W., Chiba S., Yamanaka T., Nakao T., Hara M., Tagaya I. (1979). Paralytic poliomyelitis in a child with agammaglobulinemia. Eur. J. Pediatr..

[101] Misbah S.A., Lawrence P.A., Kurtz J.B., Chapel H.M. (1991). Prolonged faecal excretion of poliovirus in a nurse with common variable hypogammaglobulinaemia. Postgrad. Med. J..

[102] Minor P. (2001). Characteristics of poliovirus strains from long-term excretors with primary immunodeficiencies. Dev. Biol..

[103] MacLennan C., Dunn G., Huissoon A.P., Kumararatne D.S., Martin J., O’Leary P., Thompson R.A., Osman H., Wood P., Minor P. (2004). Failure to clear persistent vaccine-derived neurovirulent poliovirus infection in an immunodeficient man. Lancet.

[104] Dunn G., Klapsa D., Wilton T., Stone L., Minor P.D., Martin J. (2015). Twenty-Eight Years of Poliovirus Replication in an Immunodeficient Individual: Impact on the Global Polio Eradication Initiative. PLoS Pathog..

[105] Shahmahmoodi S., Parvaneh N., Burns C., Asghar H., Mamishi S., Tabatabaie H., Chen Q., Teimourian S., Gooya M.M., Esteghamati A.R. (2008). Isolation of a type 3 vaccine-derived poliovirus (VDPV) from an Iranian child with X-linked agammaglobulinemia. Virus Res..

[106] Hidalgo S., Garcia Erro M., Cisterna D., Freire M.C. (2003). Paralytic poliomyelitis caused by a vaccine-derived polio virus in an antibody-deficient Argentinean child. Pediatr. Infect. Dis. J..

[107] Centers for Disease Control and Prevention (2008). Laboratory surveillance for wild and vaccine-derived polioviruses—worldwide, January 2007–June 2008. MMWR Morb. Mortal. Wkly. Rep..

[108] Buttinelli G., Donati V., Fiore S., Marturano J., Plebani A., Balestri P., Soresina A.R., Vivarelli R., Delpeyroux F., Martin J. (2003). Nucleotide variation in Sabin type 2 poliovirus from an immunodeficient patient with poliomyelitis. J. Gen. Virol..

[109] Tharmaphornpilas P. (2005). Vaccine-derived poliovirus, Thailand, 2003. Emerg. Infect. Dis..

[110] Parvaneh N., Shahmahmoudi S., Tabatabai H., Zahraei M., Mousavi T., Esteghamati A.R., Gooya M.M., Mamishi S., Nategh R., Kew O.M. (2007). Vaccine-associated paralytic poliomyelitis in a patient with MHC class II deficiency. J. Clin. Virol..

[111] Centers for Disease Control and Prevention (2005). Poliovirus infections in four unvaccinated children—Minnesota, August–October 2005. MMWR Morb. Mortal. Wkly. Rep..

[112] Centers for Disease Control and Prevention (2007). Update on vaccine-derived polioviruses—worldwide, January 2006-August 2007. MMWR Morb. Mortal. Wkly. Rep..

[113] Mamishi S., Shahmahmoudi S., Tabatabaie H., Teimourian S., Pourakbari B., Gheisari Y., Yeganeh M., Salavati A., Esteghamati A.-R., Gooya M.M. (2008). Novel BTK mutation presenting with vaccine-associated paralytic poliomyelitis. Eur. J. Pediatr..

[114] Driss N., Mellouli F., Ben Yahia A., Touzi H., Barbouche M.R., Triki H., Bejaoui M. (2014). Sequential asymptomatic enterovirus infections in a patient with major histocompatibility complex class II primary immunodeficiency. J. Clin. Microbiol..

[115] Centers for Disease Control and Prevention (2009). Update on Vaccine-Derived Polioviruses—Worldwide, January 2008–June 2009. MMWR Morb. Mortal. Wkly. Rep..

[116] Burgos M.E., Elkik S., Barbosa P., Oleastro M., Freire C., Parra A., Caparelli M., Sarkis C. (2010). A vaccine derived poliovirus case in an immunocompromised argentinian child. Int. J. Infect. Dis..

[117] Centers for Disease Control and Prevention (2011). Update on Vaccine-Derived Polioviruses—Worldwide, July 2009–March 2011. MMWR Morb. Mortal. Wkly. Rep..

[118] Centers for Disease Control and Prevention (2012). Update on vaccine-derived polioviruses—worldwide, April 2011–June 2012. MMWR Morb. Mortal. Wkly. Rep..

[119] Diop O.M., Burns C.C., Wassilak S.G., Kew O.M. (2014). Update on vaccine-derived polioviruses—Worldwide, july 2012–december 2013. MMWR Morb. Mortal. Wkly. Rep..

[120] Wang H.-B., Luo H.-M., Li L., Fan C.-X., Hao L.-X., Ma C., Su Q.-R., Yang H., Reilly K.H., Wang H.-Q. (2017). Vaccine-derived poliovirus surveillance in China during 2001–2013: The potential challenge for maintaining polio free status. BMC Infect. Dis..

[121] Gumede N., Muthambi V., Schoub B.D. (2012). Immunodeficiency-associated vaccine-derived poliovirus type 3 in infant, South Africa, 2011. Emerg. Infect. Dis..

[122] Diop O.M., Burns C.C., Sutter R.W., Wassilak S.G., Kew O.M. (2015). Update on Vaccine-Derived Polioviruses—Worldwide, January 2014–March 2015. MMWR Morb. Mortal. Wkly. Rep..

[123] Schubert A., Bottcher S., Eis-Hubinger A.M. (2016). Two Cases of Vaccine-Derived Poliovirus Infection in an Oncology Ward. N. Engl. J. Med..

[124] Trimble R., Atkins J., Quigg T.C., Burns C.C., Wallace G.S., Thomas M., Mangla A.T., Infante A.J. (2014). Vaccine-Associated Paralytic Poliomyelitis and BCG-osis in an Immigrant Child with Severe Combined Immunodeficiency Syndrome—Texas, 2013. MMWR Morb. Mortal. Wkly. Rep..

[125] Jorba J., Diop O.M., Iber J., Henderson E., Sutter R.W., Wassilak S.G.F., Burns C.C. (2017). Update on Vaccine-Derived Polioviruses—Worldwide, January 2016-June 2017. MMWR Morb. Mortal. Wkly. Rep..

[126] Jorba J., Diop O.M., Iber J., Henderson E., Zhao K., Sutter R.W., Wassilak S.G.F., Burns C.C. (2018). Update on Vaccine-Derived Polioviruses—Worldwide, January 2017–June 2018. MMWR Morb. Mortal. Wkly. Rep..

[127] Howard W., Moonsamy S., Seakamela L., Jallow S., Modiko F., du Plessis H., Sibiya R., Kamupira M., Maseti E., Suchard M. (2021). Sensitivity of the acute flaccid paralysis surveillance system for poliovirus in South Africa, 2016–2019. J. Med. Microbiol..

