# Supplementary material for: Immunodeficiency-Related Vaccine-Derived Poliovirus (iVDPV) Infections: A Review of Epidemiology and Progress in Detection and Management
Source: Pathogens. 2024 Dec 20;13(12):1128. doi: 10.3390/pathogens13121128 (PMC11677883; doi:10.3390/pathogens13121128)
Supplement: Supplementary file 1 [file pathogens-13-01128-s001.zip › Figure S1.pdf]

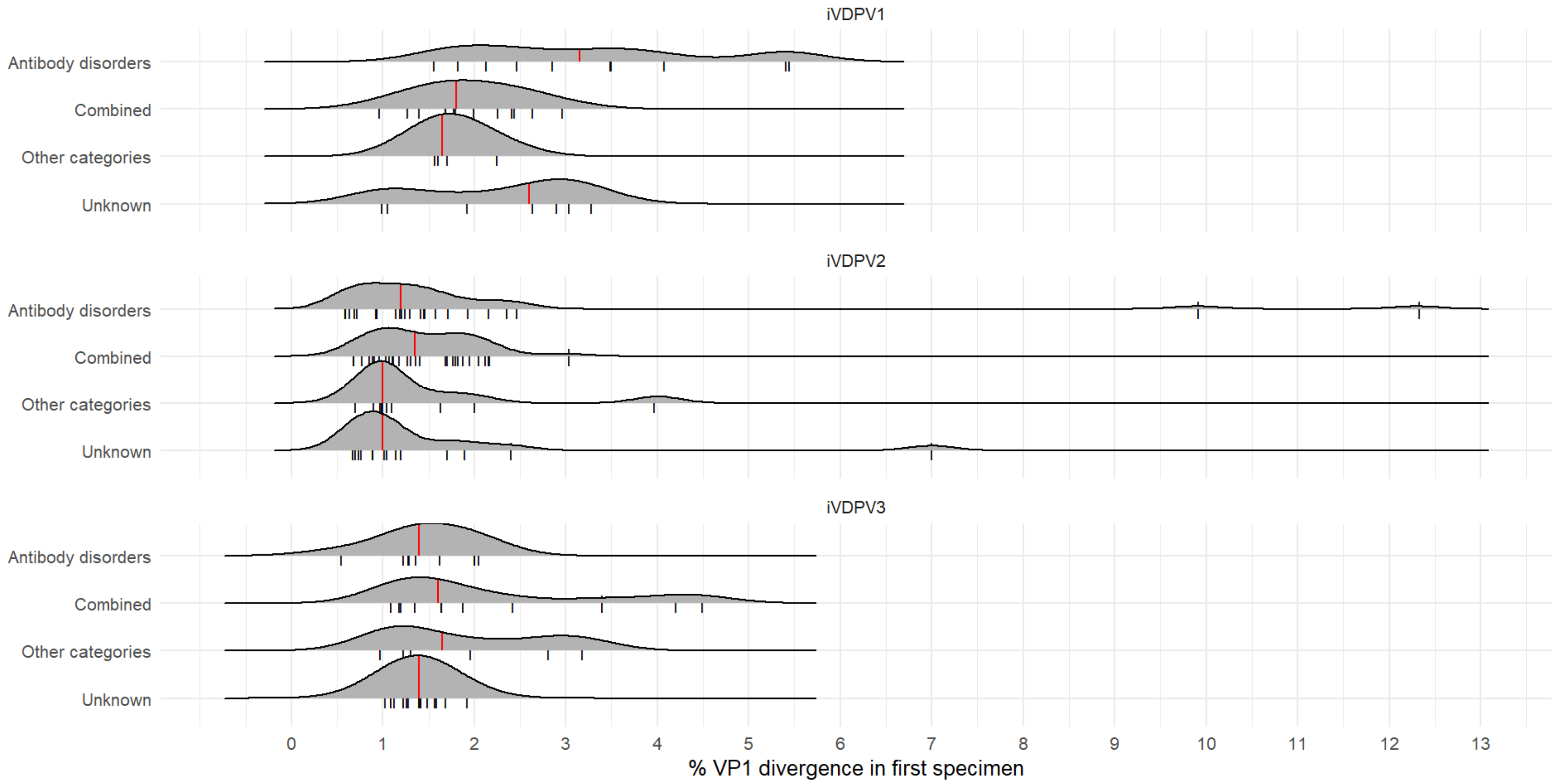


**Figure S1. VP1 divergence of isolated iVPDV from parental Sabin strains, by serotype and category of immunodeficiency.** Maximum percent VP1 divergence at the time of first specimen collection by immunodeficiency disorder by serotype. The x-axis represents the maximum percent VP1 divergence at the time of first specimen collection. Each vertical line below the x-axis represents an individual patient. Curves are a smoothed density estimate of VP1 divergence. The red lines are the median divergence by serotype and category of immunodeficiency. VP1 – viral protein 1.
