# Supplementary material for: Immunodeficiency-Related Vaccine-Derived Poliovirus (iVDPV) Infections: A Review of Epidemiology and Progress in Detection and Management
Source: Pathogens. 2024 Dec 20;13(12):1128. doi: 10.3390/pathogens13121128 (PMC11677883; doi:10.3390/pathogens13121128)
Supplement: Supplementary file 1 [file pathogens-13-01128-s001.zip › Table S1.pdf]

Supplementary Table S1. Characteristics of iVDPV cases in the WHO registry as of June 2024.

No.	Year of detection	Country	Gender	Age at detection	Immuno-deficiency disorder	Paralysis	Serotype of first specimen	Maximum VP1 divergence for first specimen	Time of excretion since detection	Outcome	References
1	1962	UK	Male	3	HGG	No	1	NA	NA	Dead	[38,39,41,61,95,96]
2	1962	UK	Female	20	HGG	No	3	NA	1.83	Dead	[38,39,61,95-97]
3	1977	Japan	Male	1.9	AGG	Yes	2	NA	2.8	Dead	[61,65,98-100]
4	1980	USA	Female	1.7	AGG	Yes	2	NA	NA	Dead	[59,61,65,95]
5	1981	USA	Male	16	CVID	Yes	1	NA	9.25	Dead	[59,61,65,95]
6	1986	USA	Male	0.9	AGG	Yes	2	NA	NA	Alive (stopped excreting)	[59,61,65,95]
7	1986	USA	Female	11	CVID	No	1	5.4	NA	Unknown	[59,61,95]
8	1987	UK	Male	34	CVID	No	2	NA	1	Alive (stopped excreting)	[59,61,65,95,96,101]
9	1989	USA	Female	0.6	AGG	Yes	1	NA	NA	Unknown	[59,61,65,95]
10	1990	USA	Female	1.3	SCID	Yes	2	NA	NA	Dead	[59,61,65,95]
11	1990	Germany	Male	7	CVID	Yes	1	5.4	6.09	Alive (stopped excreting)	[59,61,62,65,95]
12	1991	USA	Female	0.7	CVID	Yes	2	NA	NA	Dead	[59,61,65,95]
13	1995	USA	Female	0.3	SCID	Yes	2	NA	NA	Dead	[59,61,65,95]
14	1995	UK	Male	25	CVID	No	2	9.9	26.42	Alive (stopped excreting)	[39,61,64,65,95,96,102-104]
15	1995	Iran	Female	1.42	HGG	Yes	2	2.2	0.01	Dead	[61,65,95,105]
16	1998	Argentina	Male	3	AGG	Yes	1	2.8	NA	Alive (stopped excreting)	[61,65,105,106]
17	2000	Germany	Female	24	CVID	Yes	1	3.5	8.67	Dead	[65,105,107]
18	2000	UK	Male	13	CVID	No	2	NA	NA	Alive (stopped excreting)	[65,105,107]
19	2000	Italy	Female	1.67	AGG	Yes	2	0.9	0.05	Alive (stopped excreting)	[52,65,108]
20	2001	China	Missing	Missing	Unknown	Yes	3	1	NA	Unknown	WHO registry

No.	Year of detection	Country	Gender	Age at detection	Immuno-deficiency disorder	Paralysis	Serotype of first specimen	Maximum VP1 divergence for first specimen	Time of excretion since detection	Outcome	References
**21**	2001	Taiwan	Male	8	CVID	Yes	1	2.43	1.2	Alive (stopped excreting)	[42,59,61,65,95]
**22**	2002	Kazakhstan	Female	2	HGG	Yes	2	1.5	NA	Dead	[61,65]
**23**	2002	Kuwait	Female	2	MHC class II deficiency	No	2	NA	NA	Dead	[59,61,65,95]
**24**	2002	UK	Female	1.5	Other disorders	No	2	NA	NA	Alive (stopped excreting)	[39,61,65,95]
**25**	2003	Peru	Male	0.8	AGG	Yes	2	1.2	0.3	Alive (stopped excreting)	[59,61,65,95]
**26**	2003	Thailand	Male	1.5	HGG	Yes	2	1.6	0.51	Alive (stopped excreting)	[59,61,65,95,109]
**27**	2005	China	Male	2	AGG	Yes	2	1.2	1.66	Dead	[61,65]
**28**	2005	Morocco	Male	1.2	SCID	Yes	2	1.8	0.53	Dead	[61,65,77]
**29**	2005	Saudi Arabia	Missing	0.8	SCID	No	2	1.9	NA	Dead	[65]
**30**	2005	Syria	Female	0.5	HGG	Yes	2	NA	NA	Unknown	[61,65]
**31**	2005	Iran	Male	0.6	MHC class II deficiency	Yes	2	1.1	0.31	Dead	[61,65,105,110]
**32**	2005	USA	Female	0.6	SCID	No	1	2.3	0.35	Alive (stopped excreting)	[61,65,68,111]
**33**	2007	Kuwait	Female	0.67	SCID	Yes	3	1.2	0.22	Alive (stopped excreting)	[65,112]
**34**	2006	Syria	Male	0.7	Other combined immunodeficiencies	Yes	2	2.2	0.05	Dead	[65,112]
**35**	2006	Tunisia	Male	0.9	MHC class II deficiency	No	2	2	NA	Dead	[58,61,65,112]
**36**	2006	Iran	Male	0.83	SCID	Yes	2	1.7	0.11	Dead	[65,74,107]
**37**	2006	Iran	Male	1.25	AGG	Yes	3	2	0.17	Dead	[65,74,105,107,113]
**38**	2007	Belarus	Male	3	HGG	Yes	2	NA	NA	Unknown	[107]
**39**	2007	Egypt	Female	0.33	SCID	Yes	3	1.1	NA	Dead	[65,107,112]

No.	Year of detection	Country	Gender	Age at detection	Immuno-deficiency disorder	Paralysis	Serotype of first specimen	Maximum VP1 divergence for first specimen	Time of excretion since detection	Outcome	References
**40**	2007	Iran	Male	0.6	AGG	Yes	2	NA	0.98	Alive (stopped excreting)	WHO data
**41**	2007	Russia	Female	0.9	HGG	Yes	1	NA	NA	Unknown	[107]
**43**	2007	Iran	Female	0.42	SCID	Yes	1;2	2	0.06	Dead	[65,74,107]
**44**	2008	Iran	Male	1.7	AGG	Yes	2	NA	0.98	Alive (stopped excreting)	[65,113]
**45**	2009	Tunisia	Male	7.3	MHC class II deficiency	No	1	1.6	NA	Alive (stopped excreting)	[65,114]
**46**	2009	USA	Female	44	CVID	Yes	2	12.3	0.08	Dead	[65,93,115]
**47**	2009	Argentina	Male	1.3	AGG	Yes	1	3.5	0.55	Alive (stopped excreting)	[65,115,116]
**48**	2009	Colombia	Male	1.3	AGG	Yes	2	1.5	NA	Unknown	[65,117]
**49**	2009	India	Male	11	CVID	Yes	1	4.1	0.3	Dead	[65,117]
**50**	2010	Sri Lanka	Male	0.8	SCID	No	2	0.9	0.04	Dead	[65,94,117]
**51**	2010	India	Female	10	CVID	Yes	2	1.2	0.31	Alive (stopped excreting)	[117]
**52**	2010	Algeria	Female	0.5	MHC class II deficiency	Yes	2	1	0.46	Dead	[65,117]
**53**	2010	Iraq	Male	0.7	Unknown	Yes	2	1.2	0.13	Dead	[65,117]
**54**	2011	Algeria	Female	1.2	MHC class II deficiency	Yes	3	2.8	NA	Alive (stopped excreting)	WHO data
**55**	2011	Algeria	Female	0.3	MHC class II deficiency	No	3	1.2	NA	Alive (stopped excreting)	WHO data
**56**	2011	Algeria	Male	0.75	MHC class II deficiency	No	2	1	1.74	Dead	WHO data
**57**	2011	China	Male	0.58	CVID	No	2	0.9	NA	Alive (excreting at last specimen)	[117]
**58**	2011	Egypt	Female	0.7	SCID	No	2	1.4	0.02	Dead	[51,65,118]
**59**	2011	India	Male	1	HGG	Yes	2	0.7	0.46	Dead	WHO data
**60**	2011	India	Female	0.3	HGG	Yes	2	0.6	0.08	Dead	WHO data

No.	Year of detection	Country	Gender	Age at detection	Immuno-deficiency disorder	Paralysis	Serotype of first specimen	Maximum VP1 divergence for first specimen	Time of excretion since detection	Outcome	References
**61**	2011	India	Male	7	CVID	Yes	3	1.4	0.1	Alive (stopped excreting)	WHO data
**63**	2011	Sri Lanka	Female	8.4	CVID	Yes	3	1.3	0.75	Alive (stopped excreting)	[65,94,118]
**64**	2011	Turkey	Male	1	CVID	No	2	NA	NA	Unknown	[65,117-119]
**65**	2011	West Bank and Gaza Strip	Male	1	SCID	No	2	1.2	0.15	Dead	[65,118]
**66**	2011	China	Male	2.3	CVID	Yes	3	2	0.92	Dead	[65,117,120]
**67**	2011	China	Female	9	CVID	Yes	2	NA	NA	Dead	[65,117]
**68**	2011	Egypt	Male	0.5	SCID	Yes	3	4.2	0.4	Alive (stopped excreting)	[51,65,118]
**69**	2011	Egypt	Male	1.7	AGG	Yes	1	2.1	0.1	Alive (stopped excreting)	[51,65,118]
**70**	2011	Iran	Male	0.7	Other disorders	Yes	2	1	1.29	Dead	[65,118]
**71**	2011	Iran	Male	1.3	AGG	Yes	2	2.44	0.44	Alive (stopped excreting)	[65,118]
**72**	2011	South Africa	Male	0.8	Other combined immunodeficiencies	Yes	3	1.9	0.19	Alive (stopped excreting)	[65,118,121]
**73**	2011	Iran	Male	2.1	SCID	Yes	1;2	3	0.08	Dead	[65,118]
**74**	2012	Egypt	Male	0.33	SCID	Yes	1;2	NA	NA	Dead	WHO data
**75**	2012	India	Female	0.5	HGG	Yes	2	1.7	0.56	Dead	[65,118]
**76**	2012	China	Male	0.9	CVID	Yes	2;3	1.3	0.47	Alive (stopped excreting)	[65,118]
**77**	2012	Iran	Male	0.5	SCID	Yes	2	2.1	0.17	Dead	[65,73,118]
**78**	2012	Egypt	Female	0.5	SCID	No	2	1.1	NA	Dead	[119]
**79**	2012	Iraq	Male	2	Unknown	Yes	2	1	1.05	Dead	[119]
**80**	2012	Iran	Male	1	AGG	Yes	2	1.4	0.21	Alive (stopped excreting)	[119]

No.	Year of detection	Country	Gender	Age at detection	Immuno-deficiency disorder	Paralysis	Serotype of first specimen	Maximum VP1 divergence for first specimen	Time of excretion since detection	Outcome	References
**81**	2012	Egypt	Male	0.4	Other combined immunodeficiencies	Yes	2	1	0.42	Dead	[119]
**82**	2013	Algeria	Female	0.4	MHC class II deficiency	No	2	0.67	NA	Alive (excreting at last specimen)	WHO data
**83**	2013	China	Male	0.6	Unknown	Yes	3	1.3	0.88	Dead	[119]
**84**	2013	Egypt	Female	0.5	SCID	No	2	1.3	0.24	Dead	WHO data
**85**	2013	Libya	Female	0.4	SCID	No	2	0.9	0.28	Alive (stopped excreting)	[122]
**86**	2013	India	Male	0.6	CVID	Yes	2	1.1	0.43	Dead	[119]
**87**	2013	India	Male	0.8	HGG	Yes	2	0.9	0.11	Dead	[119]
**88**	2013	Saudi Arabia	Female	2.5	MHC class II deficiency	No	2	4	0.3	Alive (stopped excreting)	[119,123]
**89**	2013	UK	Female	Missing	Unknown	Unknown	2	NA	NA	Unknown	WHO data
**90**	2013	Iran	Male	1.1	Unknown	Yes	2	0.9	0.01	Dead	[119]
**91**	2013	USA	Male	0.6	SCID	Yes	1	1.3	0.02	Dead	[119,124]
**92**	2013	Afghanistan	Male	3	Other disorders	Yes	2	0.9	0.07	Alive (stopped excreting)	[119]
**93**	2014	Iran	Male	0.8	AGG	Yes	1	1.8	0.2	Alive (stopped excreting)	[122]
**94**	2014	Tunisia	Male	11.8	MHC class II deficiency	No	2	1	0.09	Alive (stopped excreting)	[122]
**95**	2014	Iran	Male	0.7	Other antibody disorders	Yes	2	0.6	0.45	Alive (stopped excreting)	[122]
**96**	2014	Iran	Male	0.8	SCID	No	1	2.44	NA	Alive (stopped excreting)	[122]
**97**	2014	Turkey	Female	2	SCID	No	3	1.33	0.94	Alive (stopped excreting)	[122]
**98**	2014	China	Male	1.2	Unknown	Yes	3	1.4	0.1	Alive (stopped excreting)	[122]

No.	Year of detection	Country	Gender	Age at detection	Immuno-deficiency disorder	Paralysis	Serotype of first specimen	Maximum VP1 divergence for first specimen	Time of excretion since detection	Outcome	References
**100**	2015	China	Male	0.7	Unknown	Yes	2	0.78	NA	Alive (excreting at last specimen)	[72]
**101**	2014	Albania	Male	0.4	AGG	Yes	3	0.5	0.21	Alive (stopped excreting)	[122]
**102**	2015	China	Male	2.2	Unknown	Yes	2	NA	NA	Alive (excreting at last specimen)	[72]
**103**	2015	China	Female	0.5	Unknown	Yes	1	1.1	NA	Alive (excreting at last specimen)	[72]
**104**	2015	West Bank and Gaza Strip	Female	0.5	SCID	No	2	1	1.02	Alive (stopped excreting)	[40]
**105**	2015	India	Female	2	CVID	Yes	2	2.4	0.5	Alive (stopped excreting)	[72]
**106**	2015	Iran	Female	0.5	SCID	Yes	2	0.9	0.53	Dead	[72]
**107**	2015	Iraq	Female	0.75	Unknown	Yes	2	1.7	0.02	Dead	[72]
**108**	2015	Iran	Male	0.25	SCID	No	2	0.7	0.12	Dead	[72]
**109**	2015	Iran	Female	1	SCID	No	2	1.1	0.08	Dead	[72]
**110**	2015	Iran	Male	1	SCID	No	2	1.8	NA	Alive (excreting at last specimen)	[72]
**111**	2015	Nigeria	Female	0.08	Unknown	Yes	2	NA	0.05	Dead	[72]
**112**	2015	Iran	Female	0.8	SCID	No	2	1.3	NA	Alive (excreting at last specimen)	[72]
**113**	2015	Oman	Male	0.7	MHC class II deficiency	No	2	1.6	0.4	Dead	[72]
**114**	2015	Algeria	Male	0.75	Other combined immunodeficiencies	No	2	1.7	NA	Dead	[72]
**115**	2015	Algeria	Male	1.8	Unknown	Yes	3	1.56	NA	Alive (excreting at last specimen)	WHO data
**116**	2016	Egypt	Male	1	SCID	No	2	1.4	0.61	Alive (stopped excreting)	[125]

No.	Year of detection	Country	Gender	Age at detection	Immuno-deficiency disorder	Paralysis	Serotype of first specimen	Maximum VP1 divergence for first specimen	Time of excretion since detection	Outcome	References
**117**	2016	India	Male	5.3	AGG	Yes	2	0.6	0.11	Alive (stopped excreting)	[125]
**118**	2016	Iraq	Female	0.6	Unknown	Yes	2	0.7	NA	Dead	[72]
**119**	2015	Turkey	Missing	Missing	Unknown	No	3	NA	NA	Unknown	[72]
**120**	2015	Turkey	Missing	Missing	Unknown	Yes	2	0.7	NA	Unknown	[72]
**121**	2016	Argentina	Male	0.9	AGG	No	2	0.66	0.31	Alive (stopped excreting)	[125]
**122**	2016	Egypt	Female	0.6	SCID	No	2	0.66	NA	Alive (stopped excreting)	[125]
**123**	2016	Pakistan	Male	0.6	Unknown	Yes	2	1.1	NA	Unknown	[125]
**124**	2016	Tunisia	Female	0.6	MHC class II deficiency	Yes	3	1	NA	Dead	[125]
**125**	2016	West Bank and Gaza Strip	Male	0.7	SCID	No	2	0.8	1.75	Dead	[125]
**126**	2016	Nigeria	Male	2	Unknown	Yes	2	0.9	NA	Unknown	[125]
**127**	2017	Egypt	Male	1	SCID	Yes	2	1.9	0.03	Dead	[125]
**128**	2017	Iran	Male	1.2	Unknown	No	3	1.3	NA	Alive (excreting at last specimen)	[126]
**129**	2017	Turkey	Female	0.4	Other combined immunodeficiencies	No	3	NA	NA	Unknown	WHO data
**130**	2015	India	Male	4	SCID	No	3	4.5	2.13	Dead	[55,72]
**131**	2015	Egypt	Male	1	AGG	Yes	2	1.9	0.21	Alive (stopped excreting)	[72]
**132**	2013	India	Male	0.9	CVID	Yes	2	0.7	0.04	Alive (stopped excreting)	[122]
**133**	2017	Egypt	Male	1.3	AGG	Yes	3	2	4.92	Alive (stopped excreting)	[125]
**134**	2017	Egypt	Female	2	SCID	Yes	1	2.4	NA	Dead	NA

No.	Year of detection	Country	Gender	Age at detection	Immuno-deficiency disorder	Paralysis	Serotype of first specimen	Maximum VP1 divergence for first specimen	Time of excretion since detection	Outcome	References
**135**	2017	China	Male	1	Unknown	Yes	3	1.5	NA	Alive (excreting at last specimen)	[126]
**136**	2018	Colombia	Female	11	Unknown	Yes	1	1	NA	Alive (stopped excreting)	[126]
**137**	2018	Iran	Female	1.1	Unknown	Yes	1	2.9	NA	Alive (excreting at last specimen)	WHO data
**138**	2018	Egypt	Male	0.8	Other combined immunodeficiencies	No	1	1.67	0.46	Alive (stopped excreting)	[66]
**139**	2018	China	Male	1	Unknown	Yes	3	1.1	NA	Unknown	[66]
**140**	2018	China	Male	0.8	Unknown	Yes	3	1.4	NA	Unknown	[66]
**141**	2018	South Africa	Male	0.22	AGG	Yes	3	1.22	NA	Alive (stopped excreting)	WHO data
**142**	2018	Egypt	Female	1.3	Other combined immunodeficiencies	No	3	1.6	0.17	Alive (stopped excreting)	[66]
**143**	2017	West Bank and Gaza Strip	Female	0.5	SCID	No	3	1.2	0.18	Alive (stopped excreting)	WHO data
**144**	2018	Egypt	Male	0.8	MHC class II deficiency	No	1	1.7	NA	Alive (excreting at last specimen)	[66]
**145**	2018	Egypt	Female	1	SCID	Yes	1	2.6	0.13	Dead	[66]
**146**	2018	Egypt	Female	0.8	SCID	No	1	1.4	NA	Alive (excreting at last specimen)	[66]
**147**	2018	Egypt	Male	1.2	SCID	No	3	1.6	0.13	Dead	WHO data
**148**	2018	Iran	Female	0.6	SCID	No	1	1	NA	Dead	[66]
**149**	2018	Iran	Male	1	Other antibody disorders	Yes	1	1.6	NA	Alive (excreting at last specimen)	[66]
**150**	2019	Egypt	Female	0.9	Unknown	No	3	1.4	NA	Alive (excreting at last specimen)	[66]
**151**	2019	Tunisia	Male	0.75	MHC class II deficiency	Yes	3	1.3	1.02	Alive (stopped excreting)	[66]

No.	Year of detection	Country	Gender	Age at detection	Immuno-deficiency disorder	Paralysis	Serotype of first specimen	Maximum VP1 divergence for first specimen	Time of excretion since detection	Outcome	References
**152**	2019	Iran	Female	0.6	Unknown	Yes	1	NA	NA	Alive (excreting at last specimen)	[66]
**153**	2019	Philippines	Male	5.3	Unknown	No	2	7	3.37	Dead	[66]
**154**	2019	Egypt	Female	0.9	Unknown	No	3	1.4	NA	Alive (stopped excreting)	[66]
**155**	2019	Egypt	Female	1.2	Unknown	No	1	3	NA	Alive (excreting at last specimen)	[66]
**156**	2019	Egypt	Male	0.8	Unknown	No	1	1.9	NA	Unknown	[66]
**157**	2020	Egypt	Male	0.4	HGG	No	3	1.6	1.26	Alive (stopped excreting)	WHO data
**158**	2020	Egypt	Male	0.8	MHC class II deficiency	No	3	2	NA	Alive (excreting at last specimen)	WHO data
**159**	2018	Argentina	Female	0.75	Unknown	Unknown	3	1.1	NA	Alive (stopped excreting)	[66]
**160**	2022	China	Male	1.1	SCID	No	3	2.4	0.18	Dead	[89]
**161**	2022	India	Male	0.8	SCID	No	1	1.8	0.29	Dead	[56]
**162**	2020	India	Male	3	Other combined immunodeficiencies	No	1	1.8	0.22	Alive (stopped excreting)	WHO data
**163**	2022	India	Male	5.2	Other combined immunodeficiencies	No	1	1.7	NA	Alive (stopped excreting)	WHO data
**164**	2022	Egypt	Female	0.6	SCID	No	2	1.1	1.23	Alive (stopped excreting)	WHO data
**166**	2021	Israel	Female	2.3	MHC class II deficiency	Yes	1	2.2	NA	Unknown	WHO data
**167**	2016	Iran	Male	1.17	Unknown	Yes	2	0.67	NA	Unknown	[125]
**168**	2017	South Africa	Female	1.4	Unknown	Yes	3	1.6	NA	Alive (excreting at last specimen)	WHO data
**169**	2018	Egypt	Female	0.9	Unknown	Yes	1	2.6	NA	Dead	[66]
**170**	2018	Iran	Male	1.6	Unknown	Yes	1	3.3	NA	Alive (excreting at last specimen)	

No.	Year of detection	Country	Gender	Age at detection	Immuno-deficiency disorder	Paralysis	Serotype of first specimen	Maximum VP1 divergence for first specimen	Time of excretion since detection	Outcome	References
**173**	2023	Egypt	Female	0.8	Unknown	No	3	1.2	1.25	Alive (stopped excreting)	WHO data
**174**	2019	UK	Male	2.7	Other disorders	No	1	1.6	NA	Alive (stopped excreting)	[69]
**175**	2022	Egypt	Missing	1	Unknown	No	2	1	1.42	Dead	WHO data
**176**	2019	UK	Male	2	MHC class II deficiency	No	3	3.2	NA	Alive (stopped excreting)	[69]
**177**	2018	South Africa	Missing	Missing	MHC class II deficiency	Yes	3	NA	0.41	Dead	[127]
**178**	2017	Egypt	Male	0.9	Unknown	Yes	2	1.9	NA	Dead	WHO data
**179**	2017	Israel	Missing	Missing	Unknown	No	2	2.4	NA	Unknown	[72]
**180**	2016	Israel	Missing	Missing	SCID	No	2	1.8	NA	Unknown	[126]
**181**	2015	Iran	Missing	Missing	SCID	No	2	2.2	NA	Dead	[72]
**182**	2023	Poland	Missing	Missing	Unknown	Unknown	3	NA	NA	Alive (excreting at last specimen)	WHO data
**183**	2023	Algeria	Missing	Missing	Unknown	Unknown	1	NA	NA	Alive (excreting at last specimen)	NA
**184**	2024	China	Male	7.7	Unknown	Unknown	1;3	NA	NA	Alive (excreting at last specimen)	WHO data
**185**	2024	India	Missing	Missing	Unknown	Unknown	3	NA	NA	Alive (excreting at last specimen)	WHO data
**186**	2024	India	Missing	Missing	Unknown	Unknown	3	NA	NA	Alive (excreting at last specimen)	WHO data
**187**	2022	Iran	Missing	Missing	Unknown	Unknown	3	NA	NA	Alive (excreting at last specimen)	NA
**188**	2024	West Bank and Gaza Strip	Male	Missing	Unknown	Yes	3	1.66	NA	Alive (excreting at last specimen)	WHO data
**189**	2024	West Bank and Gaza Strip	Female	Missing	Unknown	No	3	1.9	NA	Alive (excreting at last specimen)	WHO data

**190**	2023	China	Male	6.3	SCID	Unknown	3	3.4	0.46	Dead	WHO data

AGG , agammaglobulinemia; HGG, hypogammaglobulinemia; CVID, common variable immunodeficiency disorder; MHC major histocompatibility complex; SCID, severe combined immunodeficiency disorder.   VP1 – viral protein 1. Age of detection and time of excretion since detection expressed in years.
